# Iron, Copper, and Zinc Dyshomeostasis in Cardiovascular and Cerebrovascular Diseases: Redox Mechanisms, Evidence Levels, and Translational Prospects

**DOI:** 10.3390/ijms27188053

**Published:** 2026-09-10

**Authors:** Kai Wang, Yongchao Liu, Xiaomin Li, Lingling Li, Rui Zhou

**Affiliations:** 1 School of Traditional Chinese Medicine, Beijing University of Chinese Medicine, Beijing 102488, China; hello_wkwangkai@163.com (K.W.);; 2 Medical Experimental Research Center, China Academy of Chinese Medical Sciences, Beijing 100102, China

**Keywords:** iron, copper, zinc, cardiovascular and cerebrovascular diseases, metal dyshomeostasis, oxidative stress, ferroptosis, cuproptosis

## Abstract

Iron (Fe), copper (Cu), and zinc (Zn) are essential trace elements for cardiovascular and cerebrovascular homeostasis, acting as enzymatic cofactors in energy metabolism and antioxidant defence. However, disruption of metal homeostasis can amplify oxidative injury through direct Fe/Cu redox chemistry or indirect Zn-dependent pathways. Ferroptosis and cuproptosis have been investigated as context-dependent mechanisms in various cardiovascular and cerebrovascular diseases (CCVDs), such as atherosclerosis, ischaemic/haemorrhagic stroke, diabetic vascular disease, and abdominal aortic aneurysm. This review summarizes the physiological regulation of metal homeostasis and examines pathways through which metal dyshomeostasis may contribute to vascular and neural injury, including oxidative biomolecular modification, mitochondrial dysfunction and inflammatory signalling. It further summarizes therapeutic strategies targeting metal metabolism, such as metal chelators, trace element supplementation and nanomedicine-based targeted delivery systems, which have shown protective effects predominantly in experimental models by restoring metal homeostasis and scavenging reactive oxygen species. Finally, the review highlights key challenges in clinical translation, such as tissue-specific metal detection and targeted inhibitor development, and emphasizes that precise regulation of metal metabolism may offer therapeutic opportunities for CCVDs.

## 1. Introduction

Iron (Fe), copper (Cu), and zinc (Zn) are essential trace elements that contribute to homeostasis in the cardiovascular and cerebrovascular systems. Fe and Cu are redox-active transition metals, whereas Zn is a redox-inert d-block metal whose effects on redox homeostasis are mediated mainly through protein binding and signalling. Serving as cofactors for multiple key enzymes, they play central roles in cellular metabolism, antioxidant defence, and signal transduction. For instance, Cu and Zn constitute the active centres of superoxide dismutase (SOD), with Cu mediating redox catalysis and Zn contributing primarily to structural stability, thereby supporting reactive oxygen species (ROS) scavenging [1,2]. Fe, meanwhile, forms the basis of mitochondrial respiratory chain complexes and heme synthesis, being crucial for energy metabolism in cardiomyocytes [3,4]. This review focuses on Fe, Cu, and Zn because they combine a substantial cardiovascular and cerebrovascular evidence base with complementary redox properties. Selenium and manganese also have important antioxidant and metalloenzyme functions but fall outside the primary scope because their distinct transport and enzyme systems would require separate treatment. However, a narrow window exists between the physiological benefits and pathological toxicity of metal ions. Environmental exposure to toxic metals has been associated with increased cardiovascular disease (CVD) risk [5,6]. Endogenous Fe/Cu/Zn dyshomeostasis represents a distinct mechanistic context and is considered separately in this review. Throughout this review, pathological metal-associated injury refers primarily to the expansion or redistribution of chemically reactive labile or exchangeable metal pools, rather than to total metal content, which may remain relatively inert when sequestered in ferritin, metallothioneins, or other tightly bound stores. One important mechanism linking endogenous Fe/Cu dyshomeostasis to tissue injury is oxidative stress, particularly when labile redox-active metal pools expand. When intracellular labile iron or copper pools expand owing to altered metal uptake, storage, trafficking, or export, redox-active Fe and Cu can catalyse the production of highly reactive hydroxyl radicals through Fenton or Fenton-like chemistry [7,8]. This metal-dependent ROS generation mechanism not only directly damages deoxyribonucleic acid (DNA), proteins, and lipid membranes but also forms a vicious positive feedback loop. For instance, in cerebral ischemia-reperfusion injury, abnormal intracellular zinc accumulation can synergistically enhance reduced nicotinamide adenine dinucleotide phosphate (NADPH) oxidase-induced ROS production, dramatically amplifying neuronal damage [9]. 

Disrupted metal ion metabolism exhibits profound epidemiological and pathological associations with the onset and progression of cardiovascular and cerebrovascular diseases. During atherosclerosis (AS) progression, elevated non-transferrin-bound iron (NTBI) leads to iron deposition in the vascular media, accelerating plaque progression by inducing endothelial dysfunction and foam cell formation [10,11]. Research indicates that impairment of the solute carrier family 7 member 11 (SLC7A11)/glutathione/glutathione peroxidase 4 (GPX4) axis in vascular smooth muscle cells (VSMCs) induces iron-dependent lipid peroxidation, subsequently triggering vascular calcification [12]. Furthermore, abnormal copper ion transport (e.g., STEAP4-mediated increased uptake) has been demonstrated to cause diabetic endothelial dysfunction by activating oxidative stress and inflammatory responses [13]. In cardiac diseases, both iron deficiency-induced myocardial mitochondrial dysfunction [14] and catecholamine-stimulated iron overload [15] promote heart failure and myocardial remodeling. Notably, ferroptosis—a form of iron-dependent, lipid-peroxidation-driven regulated cell death—has been implicated in diabetic cardiomyopathy [16,17] and ischemic heart disease [18,19]. In stroke, disruption of cerebral iron homeostasis has also been implicated in tissue injury. Following hemorrhagic strokes (e.g., intracerebral hemorrhage and subarachnoid hemorrhage), hemoglobin and free iron released from ruptured red blood cells can contribute to secondary brain injury, cerebral edema, and cognitive impairment [20,21,22]. In ischemic stroke, alterations in transferrin saturation and intracellular iron deposition have been associated with greater infarct size and neurological deficits [23,24].

**Figure 1:**
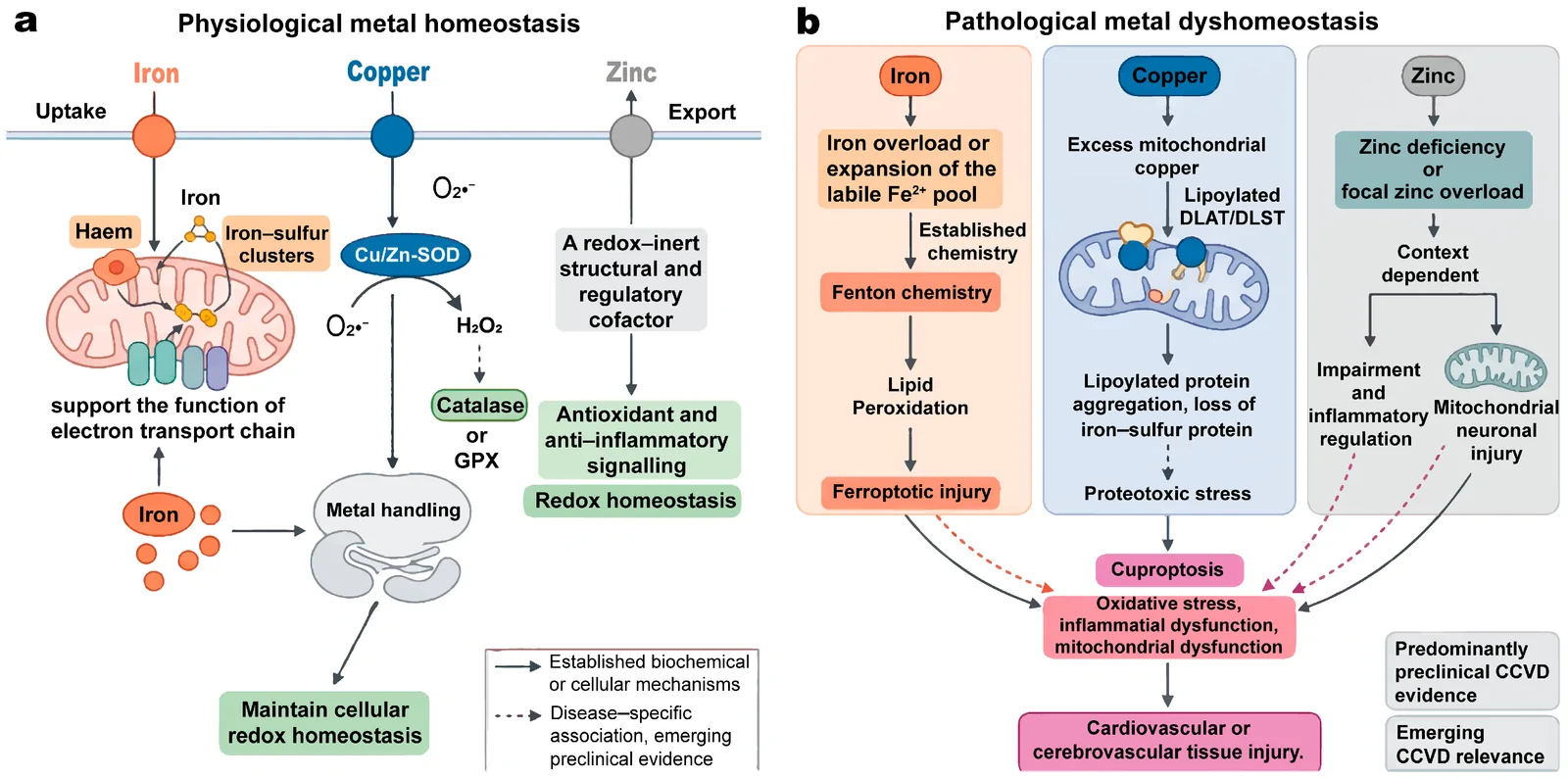
Physiological metal homeostasis and pathological metal dyshomeostasis in cardiovascular and cerebrovascular diseases. (**a**) Under physiological conditions, iron is incorporated into haem and iron–sulfur clusters to support mitochondrial electron transport, whereas copper and zinc serve as cofactors for Cu/Zn-superoxide dismutase (Cu/Zn-SOD), which converts superoxide (O_2_•^−^) to hydrogen peroxide (H_2_O_2_). H_2_O_2_ is subsequently removed by catalase or glutathione peroxidase (GPx). Zinc additionally acts as a redox-inert structural and regulatory cofactor supporting antioxidant and anti-inflammatory signalling. Coordinated metal uptake, incorporation, intracellular trafficking, storage, and export maintain cellular redox homeostasis. (**b**) Pathological iron accumulation expands the labile Fe^2+^ pool and promotes Fenton chemistry, lipid peroxidation, and ferroptotic injury. Excess mitochondrial copper binds lipoylated dihydrolipoamide S-acetyltransferase and dihydrolipoamide S-succinyltransferase (DLAT/DLST), promoting lipoylated protein aggregation, iron–sulfur protein loss, proteotoxic stress, and cuproptosis. Zinc deficiency or focal zinc overload produces context-dependent disruption of antioxidant and inflammatory regulation or mitochondrial and neuronal injury. These disturbances converge on oxidative stress, inflammation, mitochondrial dysfunction, and cardiovascular or cerebrovascular tissue injury. Solid arrows indicate established biochemical or cellular mechanisms, whereas dashed arrows indicate disease-specific associations or emerging preclinical evidence. CCVD, cardiovascular and cerebrovascular disease. Original figure created by the authors.

Given the often suboptimal efficacy of traditional antioxidant therapies in clinical settings, targeting metal ion metabolism and its mediated novel cell death pathways has emerged as a potential approach for intervening in cardiovascular and cerebrovascular diseases. Recent research has shifted beyond mere ROS scavenging to focus on blocking metal toxicity at its source or regulating metal homeostasis. For instance, biomimetic nanoenzymes (such as manganese dioxide nanoenzymes and selenium-doped copper nanoenzymes) that mimic SOD and catalase (CAT) activity not only efficiently scavenge ROS but also regulate intracellular metal ion levels, showing beneficial effects in experimental atherosclerosis and heart failure models [25,26]. For stroke, novel nanoscale scavengers and drug delivery systems have been engineered to simultaneously clear excess Fe^2+^ and suppress inflammatory responses [27,28]. Furthermore, modulating specific molecular switches—such as activating the nuclear factor erythroid 2-related factor 2 (Nrf2) pathway to enhance anti-ferroptosis defences [29,30] or leveraging lysosomal zinc ion release to block macrophage pyroptosis [31]—has demonstrated preclinical therapeutic potential. Accordingly, this review asks how Fe, Cu, and Zn dyshomeostasis alters redox homeostasis in cardiovascular and cerebrovascular diseases, what level of evidence supports each proposed mechanism, and which metal-directed interventions have credible human translational support. Evidence is considered across in vitro studies, animal models, human tissue or observational studies, and interventional clinical data. Figure 1 summarizes the mechanistic framework.

## 2. Physiological Homeostasis

### 2.1. Iron: Mitochondrial Energy Metabolism and Post-Transcriptional Fine-Tuning

#### 2.1.1. Iron–Sulphur (Fe–S) Clusters and the Mitochondrial Respiratory Chain

Iron supports mitochondrial energy metabolism and respiratory-chain function primarily through Fe–S clusters and haem. The assembly and functional integrity of mitochondrial respiratory chain complexes are highly dependent on the biosynthesis and transfer of Fe-S clusters. Research indicates that Complex I (reduced nicotinamide adenine dinucleotide (NADH) dehydrogenase), Complex II (succinate dehydrogenase), and Complex III (cytochrome bc_1_ complex) all contain Fe-S clusters. Iron deficiency specifically reduces the activity of these three complexes, thereby diminishing adenosine triphosphate (ATP) production and respiratory reserve capacity, without significantly affecting the heme-dependent Complex IV (cytochrome c oxidase) and Complex V [32]. Multiple proteins coordinate Fe–S-cluster biogenesis, transfer, and respiratory-chain assembly; disruption of these processes can impair mitochondrial respiration and promote metabolic and oxidative dysfunction [33,34,35,36]. Notably, the Complex I core subunit NDUFS2 is required for acute hypoxia sensing in pulmonary arterial smooth muscle cells. Changes in mitochondria-derived ROS, particularly H_2_O_2_, link oxygen availability to ion-channel activity and hypoxic pulmonary vasoconstriction [37,38].

Beyond Fe–S clusters, the synthesis and utilization of heme equally govern the metabolic and functional homeostasis of endothelial cells. The serine synthesis pathway (SSP) maintains intracellular serine availability in endothelial cells through phosphoglycerate dehydrogenase (PHGDH), which supports haem biosynthesis. Haem depletion impairs electron-transport-chain activity, leading to mitochondrial dysfunction, oxidative stress, and ultimately endothelial cell death [39]. During neurodevelopment, erythroblasts can even supply heme-rich mitochondrial substrates to neural epithelial cells via phagocytosis, thereby activating the electron transport chain and promoting neurogenesis [40].

At the post-transcriptional level, the iron regulatory protein (IRP) system contributes to mitochondrial function and metabolic regulation. IRP2 deficiency increases hypoxia-inducible factor-1α (HIF-1α) and hypoxia-inducible factor-2α (HIF-2α) expression; HIF-1α enhances glycolytic gene expression, whereas HIF-2α suppresses genes involved in iron–sulphur (Fe–S) cluster biogenesis and the electron transport chain, shifting cellular metabolism away from oxidative phosphorylation (OXPHOS) towards greater reliance on glycolysis [41]. This metabolic shift is particularly critical in endothelial cells. For instance, PFKFB3-driven abnormal glycolysis hijacks glucose flux in the pentose phosphate pathway, reducing cytoplasmic NADPH production and impairing mitochondrial Fe-S cluster biosynthesis, ultimately causing respiratory dysfunction and endothelium-to-mesenchymal transition (EndoMT) [42]. MicroRNAs (e.g., miR-210) also participate in this process by downregulating the Fe-S cluster chaperone ISCU, thereby inhibiting mitochondrial respiration and increasing mitochondrial reactive oxygen species (mtROS), which subsequently impairs calcium signaling in VSMCs [43].

In vascular physiology, iron and heme serve not only as cofactors for energy metabolism but also as core components of the endothelial nitric oxide synthase (eNOS) and soluble guanylate cyclase (sGC) signaling pathways. Iron-deficient diets reduce the activity of the eNOS/sGC/cyclic guanosine monophosphate (cGMP)-dependent protein kinase (PKG) pathway in mice after myocardial infarction, exacerbating oxidative/nitrosative stress [44,45]. eNOS activity is highly dependent on the correct insertion and conformation of heme. Environmental pollutants (such as nitro-polycyclic aromatic hydrocarbons) bind to heme and alter the conformation of its propionic acid side chain, significantly inhibiting eNOS activity and reducing nitric oxide (NO) production [46]. Interestingly, low-dose NO itself can act as a signaling molecule, triggering rapid heme insertion into immature sGCβ subunits as well as eNOS, inducible nitric oxide synthase (iNOS) and neuronal nitric oxide synthase (nNOS), thereby promoting maturation of functional enzyme complexes [47,48]. Furthermore, α-globin and cytoglobin—heme proteins expressed in vascular endothelial cells—participate in regulating vascular tone and ciliary motility by binding to eNOS or modulating NO bioavailability [49,50]. eNOS signaling in red blood cells also modulates intracellular NO-heme levels and extracellular paracrine signaling, playing a critical role in cardiovascular health [51].

#### 2.1.2. Post-Transcriptional Regulation Mechanisms of the Iron Regulatory Protein/Iron-Responsive Element System

The fine-tuning of intracellular iron homeostasis primarily relies on the interaction between iron regulatory proteins (IRPs) and iron-responsive elements (IREs) within the untranslated regions (UTRs) of target gene mRNAs. IRP1 and IRP2, acting as intracellular iron sensors, exert post-transcriptional regulatory functions by recognizing stem-loop structures in the 5′-untranslated region (UTR) or 3′-UTR of mRNAs [52,53]. When IRPs bind to the 3′-UTR of *transferrin receptor 1 (TfR1)* mRNA, they protect the mRNA from nuclease degradation, thereby increasing its stability and promoting iron uptake. Conversely, when IRPs bind to the 5′-UTR of ferritin heavy chain (*FTH*) or light chain (*FTL*) mRNA, they inhibit translation by blocking ribosomal complex recruitment through steric hindrance, thereby reducing iron storage [52,54]. This bidirectional regulatory mechanism ensures that cells rapidly increase available iron by upregulating TfR1 and downregulating ferritin during iron deficiency, while reversing regulation to prevent iron overload under iron-rich conditions.

Although IRP1 and IRP2 share similar functions, their molecular mechanisms for sensing iron level changes differ significantly. Under iron-rich conditions, IRP1 binds a [4Fe-4S] cluster, undergoes a conformational change, and converts into the enzymatically active cytosolic aconitase (c-Aconitase), thereby losing its ability to bind IRE [55]. In contrast, IRP2 activity is primarily regulated by protein stability. The iron-dependent ubiquitin ligase FBXL5 (F-box and leucine rich repeat protein 5) interacts with IRP2 under iron-sufficient conditions, mediating its ubiquitination and proteasomal degradation. During iron deficiency or FBXL5 dysfunction (e.g., in nanoparticle-induced toxicity), IRP2 accumulates stably, maintaining IRP/IRE system activation. This leads to abnormal overexpression of TfR1 and suppression of ferritin expression [56]. Additional modulators and non-canonical targets of IRP/IRE regulation have also been reported [57,58], but these peripheral mechanisms are outside the primary scope of this review.

Ultimately, the core physiological significance of the IRP/IRE system lies in its stringent regulation of the cellular labile iron pool (LIP). By finely tuning the balance between uptake and storage, this system maintains redox-active free iron at extremely low levels, thereby preventing the Fenton reaction at its source [56]. When this regulatory mechanism fails—such as through abnormal stabilization of IRP2—elevated LIP levels can trigger pathological processes including mitochondrial dysfunction, lipid peroxidation, and ferroptosis [55,56].

### 2.2. Copper: Extracellular Antioxidant Defence and Matrix Cross-Linking

#### 2.2.1. Copper-Binding Proteins and the Activation of the SOD Enzyme System

The SOD family serves as the first line of defence against ROS damage, with its activity highly dependent on the proper assembly of copper ions. This process is precisely regulated by specific copper chaperone proteins. Within cells, the copper chaperone CCS (Copper Chaperone for SOD) plays a crucial role as a “delivery agent.” CCS specifically recognizes and binds copper ions, transporting them to copper/zinc superoxide dismutase 1 (SOD1; Cu/Zn-SOD) in the cytoplasm [59]. This protein-protein interaction not only facilitates the formation of disulphide bonds within the SOD1 molecule, enabling it to adopt a stable folded conformation, but more importantly activates the catalytic centre of SOD1, endowing it with the ability to dismutate superoxide anion (O_2_•^−^) into H_2_O_2_ [59,60,61]. Recent studies indicate that mimicking the bimetallic active site structure of Cu/Zn-SOD to construct single-atom or bimetallic nanozymes with SOD-like activity (e.g., Cu1-N4, Cu/Zn-metal–organic framework (MOF)) effectively scavenges intracellular ROS and suppresses inflammatory responses. These findings provide experimental support for the contribution of copper-containing catalytic sites to antioxidant activity [60,62]. Notably, in senescent cells, downregulation of the copper-transporting P-type ATPase 7A (ATP7A) impedes copper efflux. The resulting abnormal copper accumulation enhances antioxidant defences by upregulating SOD1 and glutathione (GSH), yet this is frequently accompanied by impaired autophagy and disrupted cellular homeostasis [63]. For vascular walls, the function of extracellular superoxide dismutase (SOD3/Ec-SOD) is particularly critical. Although this literature collection primarily focuses on SOD1 mechanisms and novel copper-based nanoenzyme modelling studies, based on the universal catalytic mechanism of copper ions within the SOD family, we may infer that SOD3 similarly relies on copper ion catalytic activity to scavenge O_2_•^−^ at the vascular lumen surface. This process is decisive for maintaining vascular tone: by scavenging O_2_•^−^, SOD3 prevents nitric oxide (NO) from being oxidatively quenched into highly cytotoxic peroxynitrite (ONOO^−^). The bioavailability of NO is thus preserved, sustaining vasodilation and endothelial homeostasis. Novel chiral copper-polyphenol nanoenzymes (l-phen@Cu-TA) mimic the cascade reactions of SOD and CAT, not only scavenging O_2_•^−^ but also further degrading H_2_O_2_ and ONOO^−^. This multi-antioxidant mechanism demonstrates significant potential in protecting renal and vascular microenvironments from oxidative stress damage [64]. Moreover, ATP7A participates in transmembrane copper transport and may contribute to regulation of the intracellular labile copper pool and copper delivery to secretory pathways. Research indicates that in osteosarcoma cells, excessive copper ions transported intracellularly via ATP7A induce oxidative stress and cell death (e.g., cuproptosis), suggesting that fine-tuned regulation of copper homeostasis is critical for maintaining cellular survival [65].

#### 2.2.2. The ATP7A-Lysyl Oxidase Axis and Vascular Elasticity Remodelling

ATP7A is a copper-transporting P-type ATPase localised to the trans-Golgi network (TGN), whose core function is not only to efflux excess copper but also to precisely load copper ions onto precursor lysyl oxidase (LOX). LOX is a copper-dependent amine oxidase whose catalytic activity is entirely dependent on the copper metallation process mediated by ATP7A. Only upon acquiring the copper cofactor can LOX mature and be secreted into the extracellular space [66].

Within the extracellular matrix (ECM), activated LOX catalyses the oxidative deamination of lysine residues on the side chains of collagen and elastin, yielding highly reactive aldehyde derivatives—α-aminodihydroxyhexanoic acid δ-hemialdehyde (Allysine). These aldehyde residues subsequently undergo spontaneous condensation to form stable covalent cross-links, including desmosine and isodesmosine [67]. This cross-linked structure confers the necessary tensile strength and elastic recoil force upon the vascular wall, enabling it to withstand sustained haemodynamic stress. 

Dysfunction of the ATP7A-LOX axis is closely associated with the development of vascular pathology. Studies indicate that in angiotensin II (Ang II)-induced abdominal aortic aneurysm (AAA) models, ATP7A expression is significantly downregulated, leading to reduced LOX activity. This subsequently triggers elastin cleavage, loss of VSMCs, and severe vascular inflammation, ultimately promoting aneurysm formation and rupture [66]. Conversely, overexpression of ATP7A restores LOX activity and prevents AAA development. Similarly, mutations in the *LOX* gene (e.g., p.M292R) causing protein retention in the endoplasmic reticulum and preventing copper acquisition via ATP7A in the Golgi apparatus result in LOX secretion defects and functional loss. This renders the aorta highly susceptible to dilatation and aneurysm formation under increased haemodynamic stress [68]. Environmental factors, such as exposure to microplastics during ageing, have also been found to disrupt copper homeostasis by downregulating ATP7A and LOX expression, leading to vascular structural abnormalities [69]. These findings support the importance of ATP7A-mediated copper loading for LOX activity and vascular structural integrity. Dysregulation of the ATP7A–LOX axis may therefore contribute to impaired vascular elastic remodelling and aneurysm development.

### 2.3. Zinc: The Redox Switch and Inflammation Brake

#### 2.3.1. Zinc–Metallothionein Buffering and Labile-Zinc Signalling

Zinc, the second most abundant trace element in the body after iron, plays a unique role as an “oxidative-reductive switch” in maintaining cardiovascular homeostasis. Unlike iron and copper, zinc maintains a stable +2 oxidation state under physiological conditions, thereby avoiding participation in radical-generating Fenton or Haber-Weiss reactions. This redox inertness confers zinc’s capacity as an ‘indirect’ antioxidant, exerting crucial protective effects in vascular endothelial and smooth muscle cells through displacement mechanisms and signal transduction regulation. Intracellular zinc is distributed among tightly protein-bound and compartmentalised pools, while a tightly buffered labile Zn^2+^ pool remains available for cellular signalling. Cysteine-rich metallothioneins (MTs) bind exchangeable zinc to form Zn–MT complexes that function as zinc reservoirs and redox buffers [70]. Up to 30% of the amino acid residues in MT molecules are cysteine, whose abundant thiol groups can directly scavenge ROS such as hydroxyl radicals and superoxide anions. Research confirms that zinc supplementation significantly upregulates MT expression under hypoxic/reoxygenation injury or hyperglycaemic conditions. This enhances the activity of endogenous antioxidant enzymes like heme oxygenase-1 (HO-1) and NAD(P)H quinone dehydrogenase 1 (NQO1) via the Nrf2 pathway, thereby effectively scavenging ROS and protecting vascular cells from oxidative damage [71,72]. Conversely, MT gene knockout exacerbates obesity-induced cardiac remodelling and oxidative stress, further establishing the central antioxidant role of the Zn-MT system [73].

When cells encounter oxidative stress, oxidation of cysteine thiolate groups in Zn–MT complexes can mobilise Zn^2+^ into the labile intracellular pool [70]. The resulting transient increase in labile Zn^2+^ acts as a key second messenger, activating a cascade of downstream protective signalling networks. On one hand, labile Zn^2+^ promotes Nrf2 nuclear translocation, initiating the transcription of antioxidant genes [71,74]; on the other, labile Zn^2+^ inhibits nuclear factor kappa B (NF-κB) pathway activation, reducing the secretion of pro-inflammatory factors such as tumour necrosis factor-α (TNF-α) and interleukin-1β (IL-1β), thereby exerting an ‘inflammatory brake’ effect [73,75]. For instance, zinc-doped nanomaterials regulate the vascular endothelial growth factor (VEGF) signalling pathway, promoting angiogenesis and collagen deposition while scavenging ROS, thereby accelerating the healing of infected wounds [76,77]. Moreover, zinc ion flux mediated by the zinc transporter ZIP7 is critical for mitochondrial function. Its abnormal upregulation reduces mitochondrial zinc levels, thereby inhibiting PINK1/Parkin-mediated mitochondrial autophagy and exacerbating diabetic cardiomyopathy damage [78]. Concurrently, zinc activates the ERK1/2 signalling pathway via the GPR39 receptor, thereby inhibiting osteogenic differentiation and calcification in human aortic valve interstitial cells [79]. In summary, the Zn-MT system converts oxidative stress signals into zinc signals through transient labile-zinc signalling, subsequently activating cell protection networks to maintain homeostasis in the cardiovascular and cerebrovascular systems.

#### 2.3.2. Activation of the Nrf2-Kelch-like ECH-Associated Protein 1 Pathway

The Nrf2-Kelch-like ECH-associated protein 1 (Keap1) system constitutes the core endogenous defence mechanism of vascular endothelial cells against blood flow shear stress and oxidative damage. At rest, Nrf2 is anchored in the cytoplasm by Keap1, which acts as a substrate adaptor protein to mediate Nrf2 ubiquitination and proteasomal degradation, thereby maintaining Nrf2 at low levels [80,81]. Keap1 contains chemically distinct stress-sensing residues. Its minimal Zn^2+^-sensing module comprises His225, Cys226, and Cys613. Zn^2+^ coordination by this module induces a conformational change that disrupts the geometry required for cullin 3 (Cul3)–Keap1-mediated Nrf2 ubiquitination, thereby stabilising Nrf2 and permitting its nuclear accumulation [82]. Electrophile sensing can involve other Keap1 cysteines and should be distinguished from this Zn^2+^-binding mechanism. Multiple studies confirm that exogenous zinc supplementation or the utilisation of zinc-releasing biomaterials (such as Zn-MOFs or zinc-doped nanocoatings) significantly promotes Nrf2 nuclear translocation and activates this signalling pathway [83,84]. Upon release, Nrf2 translocates to the nucleus, binds to the antioxidant response element (ARE), and initiates transcription of a series of downstream cell-protective genes. Among the most critical effector molecules are HO-1, NQO1 and glutamate–cysteine ligase catalytic subunit (GCLC) [85,86]. HO-1 exerts cytoprotective effects in several experimental injury models [87,88]. Mechanistically, HO-1 catabolises haem to biliverdin, carbon monoxide, and ferrous iron; biliverdin is subsequently converted to bilirubin by biliverdin reductase. In experimental models, pharmacological HO-1 inhibition with zinc protoporphyrin (ZnPP) has been associated with increased oxidative stress and ferroptotic injury, supporting a context-dependent protective role for HO-1 [89,90,91]. Moreover, bidirectional cross-regulation exists between the Nrf2 signalling pathway and zinc homeostasis. On one hand, zinc acts as a signalling molecule to activate Nrf2; conversely, Nrf2 activation can regulate intracellular total zinc content and the expression of zinc transporters (e.g., ZnT1) and MTs. This interaction is oxygen concentration-dependent, collectively weaving a sophisticated redox defence network [71,92]. In experimental models of diabetes or exposure to toxic metals, activation of the Nrf2/Keap1 pathway has been associated with protection against mitochondrial dysfunction and ROS accumulation [92,93].

#### 2.3.3. Tumour Necrosis Factor Alpha-Induced Protein 3–NF-κB Signalling and Regulation of NADPH Oxidase

Zinc contributes to inflammatory and redox regulation through two related mechanisms. It supports tumour necrosis factor alpha-induced protein 3 (A20)-dependent negative regulation of NF-κB signalling and can also limit NADPH oxidase-associated ROS production.

A20, encoded by tumour necrosis factor alpha-induced protein 3 (*TNFAIP3*), is an anti-inflammatory ubiquitin-editing enzyme featuring an N-terminal ovarian tumour (OTU) domain and seven C-terminal zinc finger (ZnF) domains within its molecular structure [93,94]. These zinc finger domains, particularly ZnF4 and ZnF7, are crucial for A20’s physiological functions. A20 binds to ubiquitin chains through its ZnF7 and ZnF4 domains, facilitating its recruitment to the tumour necrosis factor receptor 1 (TNFR1) signalling complex and limiting inflammatory signalling and cell death [95,96]. Loss of ZnF7 function impairs A20-mediated inhibition of macrophage necroptosis and inflammasome activation, resulting in severe arthritis [97]. Combined inactivation of ZnF4 and ZnF7 leads to multi-organ inflammation and perinatal mortality [98]. Moreover, mutations affecting the ZnF2 domain of human *TNFAIP3* result in reduced A20 protein expression and excessive NF-κB pathway activation, triggering autoinflammatory diseases [99]. Consequently, zinc serves not only as an essential cofactor for A20 protein folding and structural stability, but the integrity of its domains directly determines A20’s anti-inflammatory efficacy.

NADPH oxidases are major sources of ROS in vascular tissues. Experimental zinc restriction increased NADPH oxidase-dependent superoxide production in cardiac tissue [100], whereas zinc treatment reduced ROS generation and inflammatory cytokine expression in other experimental models [101]. In experimental pulmonary arterial hypertension, reduced extracellular zinc was associated with disease progression, while zinc supplementation reduced oxidative stress and VSMC proliferation [102]. Collectively, these findings suggest that zinc status can modulate NADPH oxidase-associated oxidative signalling, although the precise effects on oxidase assembly and upstream activation remain incompletely defined.

Zinc supplementation has been demonstrated to significantly upregulate A20 expression, thereby inhibiting NF-κB pathway activation through a negative feedback mechanism. In diabetic nephropathy models, overexpression of zinc transporter 8 (ZnT8) or zinc supplementation increased A20 abundance, subsequently inhibiting high-glucose-induced NF-κB-dependent transcriptional activity and renal tubular epithelial cell apoptosis [103]. Similarly, in intracranial aneurysm models, zinc therapy markedly suppressed macrophage infiltration and aneurysm growth by inducing A20 expression and inactivating NF-κB signalling [104]. In microglia, zinc exerts anti-neuroinflammatory effects by upregulating A20, thereby reducing p65 phosphorylation levels and downstream inflammatory cytokine expression (e.g., TNF-α, interleukin-6 (IL-6)) [101]. Moreover, organic zinc compounds (e.g., zinc protein salts) demonstrate superior efficacy in mitigating heat stress-induced intestinal barrier damage. This mechanism similarly involves A20-mediated inhibition of the NF-κB-p65/matrix metalloproteinase (MMP)-2 pathway, thereby promoting tight junction protein expression and maintaining intestinal integrity [105]. Further research indicates that A20 functions as a regulator of receptor activator of nuclear factor kappa B (RANK)-induced NF-κB signalling. Its activity is not entirely dependent on deubiquitinating enzyme activity but is highly reliant on its zinc finger domain’s ability to bind ubiquitin, thereby controlling osteoclast differentiation and maintaining bone homeostasis [106].

In summary, zinc-associated cytoprotection in these experimental models involves A20-dependent restraint of NF-κB signalling and reduced NADPH oxidase-associated ROS production, as summarised in Table 1.

## 3. Pathogenic Mechanisms

### 3.1. The Fenton Reaction and Irreversible Modification of Biomolecules

#### 3.1.1. Stoichiometry of Hydroxyl Radical Burst

Under pathological conditions, Fenton chemistry involving redox-active iron and copper is an important source of reactive radicals in biological systems. Rather than being consumed stoichiometrically, these metals undergo catalytic redox cycling; biological reductants such as ascorbate regenerate the reduced metal state and sustain H_2_O_2_ conversion to reactive products. Reaction rates and radical distributions vary with metal identity, ligand environment, pH, and concentration, with copper cycling particularly rapidly in some experimental systems [107,108,109,110,111].

Regarding the specific oxidation products and their stoichiometric characteristics in the Fenton reaction under physiological conditions, academic discourse continues to explore underlying mechanisms. Whilst conventional wisdom posits that hydroxyl radicals constitute the primary product of the reaction between ferrous ions and hydrogen peroxide, alternative perspectives suggest that in neutral physiological solutions containing bicarbonate, the principal oxidation product may be the carbonate radical anion rather than hydroxyl radicals [112,113]. Nevertheless, systematic studies employing nuclear magnetic resonance (NMR) spectroscopy coupled with hydroxyl radical scavengers have provided conclusive evidence that, under neutral pH conditions, physiologically relevant Fe^2+^-citrate complexes react with hydrogen peroxide to generate hydroxyl radicals, rather than higher-valent iron (Fe(IV)) species [114]. This finding supports the classical toxicity mechanism of the Fenton reaction within cells, where hydroxyl radical generation is predominant, emphasising the pivotal role of free or weakly bound metal ions in inducing irreversible damage to biomolecules. A concise comparison of ferroptosis and cuproptosis is provided in Table 2.

#### 3.1.2. Oxidative DNA Damage: 8-Hydroxy-2′-Deoxyguanosine Formation in DNA and Genomic Instability

Metal ion-driven hydroxyl radical bursts constitute a primary source of oxidative DNA damage. Under oxidative stress conditions, hydroxyl radicals specifically attack the C-8 position of guanine within DNA molecules, resulting in the formation of 8-hydroxy-2′-deoxyguanosine (8-OHdG) [118]. 8-OHdG serves not only as a hallmark product of ROS-mediated DNA damage but also as a potent mutagenic agent. Its primary hazard lies in inducing mismatch repair failure during DNA replication, triggering G-to-T transversion mutations that subsequently lead to genomic instability [118]. At the cellular level, the persistent accumulation of this oxidative DNA damage compromises genomic integrity, triggering p53-dependent apoptotic pathways or inducing cellular senescence. Consequently, it has been implicated in the pathogenesis of numerous age-related diseases and cancers [118].

In cardiovascular pathology, the accumulation of 8-OHdG is closely associated with the progression and instability of atherosclerotic plaques. Studies indicate that atherosclerotic plaques induced by a high-fat diet are accompanied by severe iron overload and lipid peroxidation, leading to a significant elevation in 8-OHdG levels. This occurs concurrently with the disruption of mitochondrial ultrastructure within the plaque and a surge in ROS levels [119]. Particularly in the vulnerable zone of the plaque, 8-OHdG accumulation not only reflects a locally intense oxidative environment but also undermines the stability of the plaque fibrous cap by inducing cellular senescence and apoptosis, thereby potentially contributing to plaque vulnerability and rupture [119]. Pharmacological intervention studies further confirm that modulating iron metabolism and inhibiting lipid peroxidation by activating the Nrf2/HO-1/GPX4 axis significantly reduces 8-OHdG levels within plaques. This subsequently improves mitochondrial function, reduces cellular senescence, and ultimately reverses plaque instability [119]. This evidence supports 8-OHdG as a marker of oxidative DNA damage associated with plaque pathology, while its causal contribution to plaque instability remains to be established.

#### 3.1.3. Molecular Target II: Protein Carbonylation and Calcium/Calmodulin-Dependent Protein Kinase II Oxidation

**Figure 2:**
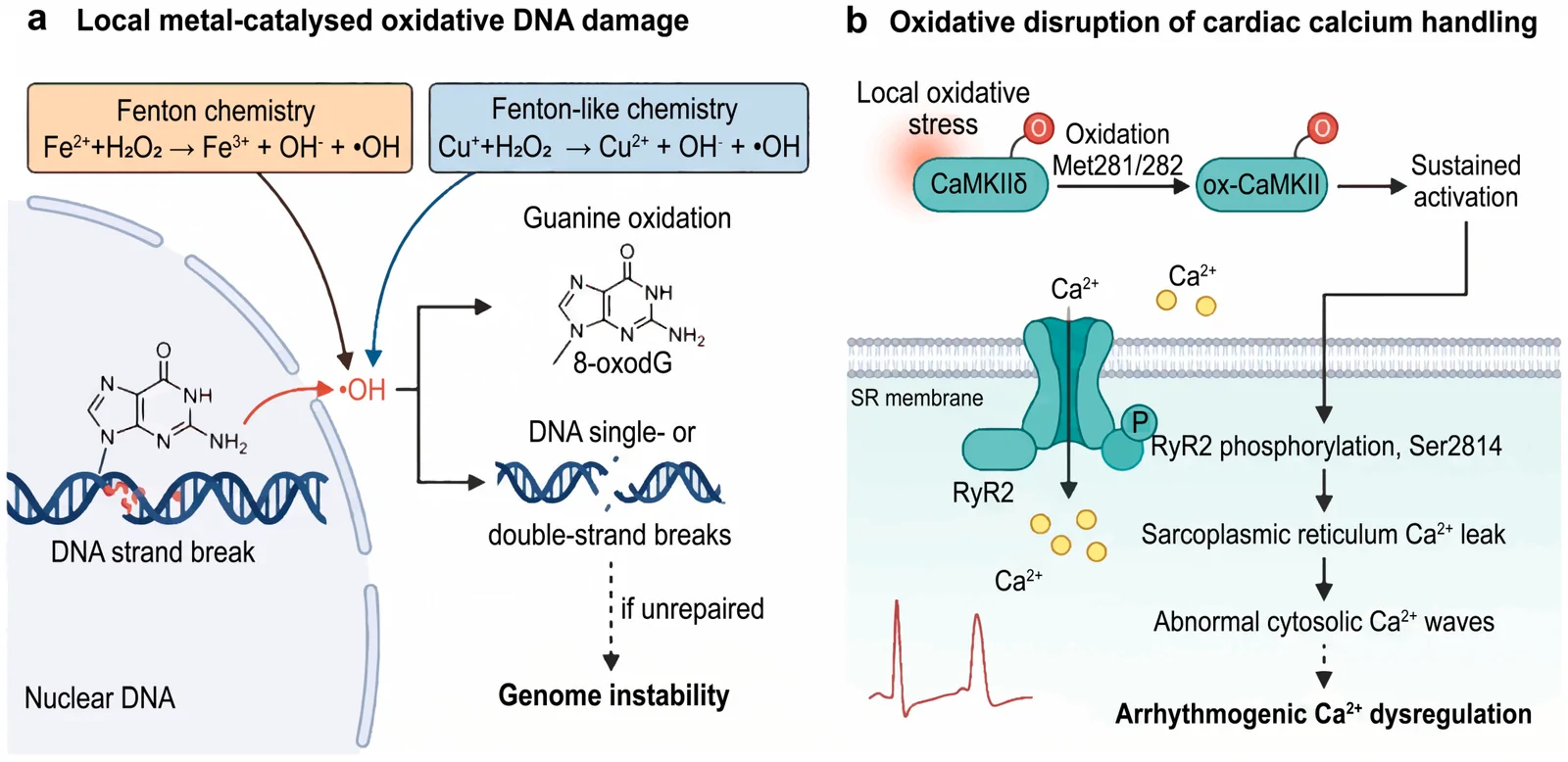
Local metal-catalysed oxidative damage to deoxyribonucleic acid (DNA) and cardiac calcium handling. (**a**) Fe^2+^ and Cu^+^ react with H_2_O_2_ through Fenton and Fenton-like chemistry, respectively, producing highly reactive hydroxyl radicals (•OH) close to their molecular targets. Local •OH generation promotes guanine oxidation to 8-oxo-7,8-dihydro-2′-deoxyguanosine (8-oxodG) and induces DNA single- or double-strand breaks. If unrepaired, these lesions may contribute to genome instability. (**b**) Local oxidative stress oxidizes the cardiac δ isoform of Ca^2+^/calmodulin-dependent protein kinase II (CaMKIIδ) at Met281/282, generating oxidized CaMKII (ox-CaMKII) and sustained kinase activation. Activated ox-CaMKII promotes phosphorylation of ryanodine receptor 2 (RyR2) at Ser2814, increasing sarcoplasmic reticulum Ca^2+^ leak and abnormal cytosolic Ca^2+^ waves and thereby contributing to arrhythmogenic Ca^2+^ dysregulation. Solid arrows indicate direct biochemical or molecular events, whereas dashed arrows denote conditional or integrated downstream outcomes. SR, sarcoplasmic reticulum. Original figure created by the authors.

As shown in Figure 2, under pathological oxidative stress conditions, ROS—particularly hydroxyl radicals, disrupt myocardial intracellular signalling networks through multiple chemical modifications. Beyond the irreversible damage caused by direct oxidation of amino acid side chains leading to protein carbonylation, ROS-mediated specific oxidative modifications of calcium/calmodulin-dependent protein kinase II (CaMKII) constitute a key molecular switch for arrhythmias. Research confirms that catecholamine stress not only elevates protein carbonylation levels but also induces oxidative activation of CaMKII [120]. This activation mechanism primarily involves oxidation at Met281 and Met282 sites within the CaMKII regulatory domain [121,122,123]. Under normal physiological conditions, CaMKII activity depends on calcium ion-calmodulin (CaM) binding; however, oxidation at Met281/282 induces a conformational change in the kinase, markedly increasing its affinity for CaM and slowing CaM dissociation by approximately threefold. This oxidative modification confers a form of ‘molecular memory’ upon CaMKII, enabling it to maintain a sustained autonomously active state even after intracellular calcium concentrations return to baseline [124].

Persistently activated oxidised CaMKII (ox-CaMKII) serves not merely as a simple signal amplifier, but as a pivotal hub linking oxidative stress to lethal arrhythmias. In early myocardial ischaemia and heart failure models, explosive increases inmitochondrial reactive oxygen species (mtROS) lead to oxidation of CaMKII Met282, subsequently causing excessive phosphorylation of ryanodine receptor 2 (RyR2) at the S2814 site [125,126]. This abnormal phosphorylation destabilises RyR2, triggering sarcoplasmic reticulum (SR) calcium leakage. This elevates diastolic cytoplasmic calcium levels and induces mitochondrial calcium overload, forming an ‘mtROS-calcium feedback loop’ that ultimately provokes lethal ventricular arrhythmias and sudden cardiac death [126]. Furthermore, ox-CaMKII phosphorylates the sodium channel Nav1.5, amplifying late sodium currents and exacerbating intracellular Na^+^/Ca^2+^ homeostasis disruption, thereby generating early afterdepolarisation (EADs) [127,128]. In ventricular myocardial tissue from atrial fibrillation (AF) patients, elevated Met281/282 oxidation levels were also observed to correlate closely with impaired systolic calcium handling and increased RyR2 phosphorylation. Crucially, this process is independent of tachycardia and is directly driven by ROS [121].

Multiple genetic engineering and pharmacological studies have established the causal role of CaMKII oxidation in pathological processes. In mouse models of Duchenne muscular dystrophy (DMD) and acute ethanol intoxication, mutating the Met281/282 site of CaMKII to oxidation-resistant valine residues (MM-VV mutation) completely abolished oxidative stress-induced SR calcium leakage and ventricular tachycardia, demonstrating that oxidation at this site is essential for arrhythmia onset [122,123]. Conversely, deficiency in methionine sulfoxide reductase A (MsrA)—an enzyme responsible for repairing oxidised methionine—exacerbates angiotensin II-induced CaMKII oxidation and atrial remodelling; supplementation with S-methyl-L-cysteine (SMLC) reverses this process by upregulating MsrA [129]. Furthermore, inhibiting ROS production via NADPH oxidase 2 (NOX2) or employing mitochondrial-targeted antioxidants effectively reduces CaMKII oxidation levels, restores calcium homeostasis, and diminishes arrhythmia incidence [126,127,130]. This evidence collectively indicates that blocking the oxidation of CaMKII at Met281/282 or enhancing its reductive repair mechanisms represents a crucial strategy for preventing and treating oxidative stress-related cardiovascular diseases.

### 3.2. Ferroptosis: Iron-Dependent Membrane Lipid Peroxidation

#### 3.2.1. Core Pathway: Collapse of the GPX4-GSH Axis and Lipid Peroxidation

Ferroptosis involves impaired antioxidant defence and the accumulation of lipid peroxides. The SLC7A11–GSH–GPX4 signalling axis is a major defence against ferroptosis, and its disruption promotes membrane lipid peroxidation [12,115].

As a molecular switch within the ferroptosis defence system, SLC7A11 serves as the core subunit of the cystine/glutamate antiporter (System Xc−), responsible for the uptake of extracellular cystine—the rate-limiting precursor for GSH [131]. Research indicates that the functional state of SLC7A11 directly determines intracellular cysteine pool capacity. In pathological models such as VSMC calcification and diabetic cardiomyopathy, high calcium-phosphate environments or high-glucose-induced DNA damage response (DDR) significantly downregulate SLC7A11 expression, leading to intracellular GSH depletion and subsequently triggering ferroptosis [12,132]. Furthermore, SLC7A11 stability is regulated by multiple post-translational modifications. For instance, the long non-coding RNA *HEPFAL* accelerates ferroptosis by promoting SLC7A11 ubiquitination and degradation [133], while the SIRT1/p53 axis modulates *SLC7A11* transcriptional activity via deacetylation to maintain redox homeostasis [134]. Notably, SLC7A11-mediated cystine uptake not only supplies precursors for GSH synthesis but also directly regulates GPX4 protein synthesis efficiency via the mTORC1-4EBP signalling axis, revealing a profound link between nutrient sensing and ferroptosis defence mechanisms [135].

GPX4 is currently the only known enzyme capable of reducing membrane-bound phospholipid hydroperoxide (L-OOH) to the non-toxic lipid alcohol (L-OH), with its activity being highly dependent on GSH as a reducing cofactor [115,136]. When SLC7A11 function is impaired or GSH synthesis is inhibited (e.g., during metabolic reprogramming in pathological states or following pharmacological intervention), GPX4 loses its activity due to cofactor deficiency, resulting in the cell’s inability to clear lipid peroxides [131,137]. Multiple studies indicate that GPX4 expression and activity undergo precise molecular regulation: in Parkinson’s disease models, the RNA-binding protein HNRNPA2B1 stabilises *GPX4* transcripts via an m6A modification recognition mechanism, with its degradation leading to reduced GPX4 levels and impaired lipid peroxidation defence [138]; In myocardial ischaemia–reperfusion injury, the guanylate cyclase 1 soluble subunit alpha 1 (GUCY1A1)–cyclic guanosine monophosphate (cGMP)–PKG pathway promotes LDHA-dependent GPX4 phosphorylation, thereby reducing chaperone-mediated autophagic degradation of GPX4 and preserving its anti-ferroptotic function [139]. Conversely, inhibition of the C-terminal domain of Hsp90 disrupts the interaction between GPX4 and the mitochondrial outer-membrane protein voltage-dependent anion channel 1 (VDAC1), exacerbating mitochondrial damage and ferroptosis [140].

The collapse of the GPX4-GSH axis directly leads to the exponential accumulation of polyunsaturated fatty acid (PUFA) peroxides on the cell membrane, constituting the final step in ferroptosis execution. Research indicates that polyunsaturated fatty acid-containing phosphatidylethanolamines (PUFA-PEs) serve as the primary substrate for lipid peroxidation, with the accumulation of its oxidised form (PE-PUFA-OOH) directly compromising membrane integrity [116,141]. In renal injury models, increased phosphor-ethanolamine synthase promotes PE-PUFA substrate generation, which rapidly converts to oxidised phospholipids (e.g., PE(18:0/20:4+3[O])) under SLC7A11/GPX4-downregulated conditions, triggering cell death [141]. Moreover, acyl-CoA synthetase long-chain family member 4 (ACSL4) acts as an accomplice in this process, responsible for enriching PUFAs and integrating them into membrane phospholipids, thereby providing the substrate basis for lipid peroxidation. In models of septic cardiomyopathy and stroke, ACSL4 upregulation frequently coincides with GPX4 downregulation, synergistically driving lethal waves of lipid peroxidation [142,143]. This oxidative damage triggered by GPX4 inactivation extends beyond the plasma membrane to involve mitochondrial membrane injury, leading to mitochondrial dysfunction and further ROS surges, thereby establishing a vicious cycle [116,144].

#### 3.2.2. Iron Metabolism Axis: Ferritinophagy and Nuclear Receptor Coactivator 4

Ferritinophagy is a major mechanism regulating intracellular iron homeostasis. Nuclear receptor coactivator 4 (NCOA4) functions as a selective cargo receptor that recognises and binds ferritin heavy chain 1 (FTH1), delivering ferritin to autophagosomes for lysosomal degradation and the release of labile Fe^2+^ [145,146]. This process undergoes multi-tiered, finely tuned molecular regulation: at the post-translational modification level, the deubiquitinating enzyme USP14 is activated during ischaemia–reperfusion (I/R) injury, maintaining NCOA4 stability by removing its ubiquitin chains, resulting in abnormally elevated NCOA4 protein levels [147]; conversely, small ubiquitin-like modifier (SUMO)-specific protease 2 (SENP2)-mediated de-SUMOylation promotes NCOA4 degradation by disrupting its interaction with the deubiquitinating enzyme OTUB1, thereby suppressing excessive ferritinophagy [148]. Furthermore, epigenetic modifications are implicated, such as the m6A reader protein YTH N6-methyladenosine RNA-binding protein 2 (YTHDF2) promoting methylation-dependent degradation of *NCOA4* mRNA [149], whilst the long non-coding RNA FOXF1 adjacent non-coding developmental regulatory RNA (*FENDRR*) enhances NCOA4 expression by upregulating its m6A modification levels, thereby driving ferroptosis [150].

Within the pathological context of stroke and I/R injury, dysregulated ferritinophagy mediated by NCOA4 constitutes one of the fundamental causes of neuronal and vascular cell death. Research confirms that the cellular stress environment during the I/R phase significantly upregulates NCOA4 expression, prompting excessive recruitment of FTH1 into lysosomes and resulting in a dramatic expansion of the intracellular ‘LIP’ [147,151]. This increase in labile Fe^2+^ catalyses the Fenton reaction, generating substantial hydroxyl radicals that directly trigger membrane lipid peroxidation. Targeting this mechanism, multiple studies have proposed intervention strategies: novel small-molecule inhibitors 9a and ginkgolide B have both been demonstrated to directly disrupt the NCOA4-FTH1 protein interaction, thereby blocking ferritin transport into lysosomes. This effectively reduces neuronal iron overload and lipid peroxidation, significantly improving infarct volume and neurological function [151,152]. In neonatal hypoxic-ischaemic brain injury models, disruption of the NCOA4-FTH1 complex similarly demonstrated marked neuroprotective effects by inhibiting lysosomal iron accumulation and maintaining the activity of the SLC7A11/GPX4 antioxidant axis [153].

Beyond acute ischaemic injury, NCOA4-driven iron metabolism dysregulation is also extensively implicated in chronic vascular pathologies such as atherosclerosis and vascular calcification. Within atherosclerotic plaques, highly expressed NCOA4 not only degrades cytoplasmic ferritin but also binds to mitochondrial ferritin (FTMT), disrupting mitochondrial iron homeostasis and inducing ferroptosis in VSMCs alongside plaque instability [154]. Furthermore, stimuli such as hyperphosphatemic environments or ionising radiation activate the p38 mitogen-activated protein kinase (p38 MAPK) signalling pathway or Lipocalin-2 (LCN2)-mediated upregulation of NCOA4, accelerating the autophagic degradation of FTH1. This leads to iron overload and phenotypic transformation within vascular endothelial cells and smooth muscle cells, ultimately promoting vascular calcification and inflammatory responses [155,156]. Activation of the innate immune pathway cyclic GMP–AMP synthase–stimulator of interferon genes (cGAS–STING) has also been found to promote NCOA4-mediated ferritinophagy, further exacerbating tissue damage [157]. Consequently, targeting the NCOA4-FTH1 axis has emerged as a key therapeutic strategy for disrupting the ferroptosis cascade in vascular diseases.

#### 3.2.3. Lipid Synthesis Axis: ACSL4-Driven Lipid Peroxidation

ACSL4 is a key determinant in ferroptosis and is regarded as the “executive” of lipid peroxidation. Its core function lies in catalysing the conversion of long-chain polyunsaturated fatty acids (particularly arachidonic acid, AA) into acyl-CoA. This acyl-CoA is subsequently re-esterified via lysophosphatidylcholine acyltransferase 3 (LPCAT3) and incorporated into cell membrane phospholipids, forming polyunsaturated phospholipids (PUFA-PLs) that are readily oxidized [158]. Given its pivotal role in ferroptosis, ACSL4 expression undergoes intricate regulation spanning transcription, translation, and post-translational modification. At the transcriptional level, the androgen receptor (AR) acts as a transcription regulator to directly inhibit *ACSL4* transcription, thereby limiting lipid peroxidation [159]; conversely, histone lactylations such as H3K18la promote *ACSL4* transcription [160]. At the post-transcriptional level, the m6A demethylase ALKBH5 maintains high ACSL4 expression by removing m6A modifications from the 3′UTR region of *ACSL4* mRNA, thereby preventing YTHDF2-mediated degradation [161]. Furthermore, interactions between the translation initiation factor eIF3 complex and ALDH2 regulate the selective translation of *ACSL4* mRNA [162]. At the protein modification level, E3 ubiquitin ligases RNF5 and Marchf6 both promote ACSL4 ubiquitin-mediated degradation [163,164], whereas SUMO-specific protease 2 (SENP2)-mediated de-SUMOylation reduces ACSL4 stability [165]. Conversely, protein kinase C (PKC)-mediated phosphorylation of ACSL4 enhances its pro-ferroptotic activity [166].

In vascular pathologies such as chronic kidney disease, diabetes mellitus, and hypertension, the abnormal upregulation of ACSL4 serves as a central driver of ferroptosis in VSMCs, thereby precipitating vascular remodelling. Studies indicate that ACSL4 expression levels correlate positively with lesion severity in aortic dissection and atherosclerotic lesions [159,167]. ACSL4-mediated ferroptosis in VSMCs not only leads to cell loss but also triggers a robust inflammatory response. Ferroptotic cells release damage-associated molecular patterns (DAMPs) such as high mobility group box protein B1 (HMGB1). These molecules bind to TLR4 receptors as danger signals, activating the NF-κB pathway to promote macrophage polarisation towards the pro-inflammatory M1 phenotype. This further activates NLR family pyrin domain-containing 3 (NLRP3) inflammasomes, establishing a vicious ‘ferroptosis-inflammation’ cycle [168,169]. Moreover, ACSL4-dependent ferroptosis directly activates the downstream pyroptosis signalling pathway, leading to the substantial release of the pro-inflammatory cytokine IL-1β, thereby exacerbating cardiac remodelling and dysfunction during the progression of heart failure [170]. In a hypertension model, the formation of neutrophil extracellular traps (NETs) further upregulates ACSL4 expression in VSMCs, inhibits GPX4, thereby driving fibrotic remodelling [169].

Activation of ACSL4 in endothelial cells constitutes a pivotal mechanism underlying vascular barrier dysfunction and increased permeability. In diabetic kidney disease (DKD), activation of the lipoprotein-associated phospholipase A2 (Lp-PLA2)/lysophosphatidylcholine (LPC) axis markedly upregulates ACSL4 in glomerular endothelial cells, leading to ROS accumulation and endothelial ferroptosis, which subsequently triggers proteinuria and renal injury [171]. The hyperglycaemia-induced DDR also upregulates ACSL4 via activation of the DNA-dependent protein kinase (DNA-PK) complex, promoting endothelial ferroptosis and microvascular dysfunction [132]. Moreover, activation of the mechanosensitive ion channel Piezo1 promotes ACSL4-dependent lipid peroxidation, causing microvascular endothelial disruption and tissue oedema in limb I/R injury [166]. In sepsis-associated acute lung injury, a hyperlactic environment upregulates endothelial ACSL4 via histone lactoylation, leading to increased pulmonary microvascular permeability [160]. Notably, ACSL4-mediated ferroptosis also constitutes a primary mechanism disrupting the integrity of the blood-brain barrier (BBB) and blood-spinal cord barrier (BSCB). In models of ischaemic stroke and spinal cord injury, inhibiting ACSL4 significantly reduces endothelial ferroptosis, preserves tight junction proteins, and thereby maintains barrier function within the central nervous system [172,173].

### 3.3. Cuproptosis: Targeted Disruption of the Mitochondrial Tricarboxylic Acid Cycle

As a form of regulated cell death only recently defined, cuproptosis exhibits molecular mechanisms distinctly different from apoptosis, pyroptosis, and ferroptosis. Its core characteristic lies in intracellular excess copper ions directly targeting mitochondrial respiratory mechanisms, causing irreversible damage to the tricarboxylic acid cycle (TCA cycle) and its associated enzyme systems, thereby triggering protein toxicity stress and cell death [174,175]. Unlike ferroptosis, which primarily relies on lipid peroxidation, cuproptosis is highly dependent on the cellular mitochondrial respiratory state. This renders tissues heavily reliant on OXPHOS for energy supply—such as the heart and brain—particularly susceptible to this process [176,177].

#### 3.3.1. Molecular Mechanism: Abnormal Protein Lipoylation Mediated by Ferredoxin 1

Ferredoxin 1 (FDX1) has been demonstrated to be a pivotal upstream regulatory enzyme within the cuproptosis pathway. FDX1 not only participates in mitochondrial metabolism but, crucially, regulates the valence state of copper ions, reducing Cu^2+^ within mitochondria to Cu^+^, which exhibits heightened biological toxicity and reactivity [176,178]. Research indicates that FDX1 expression levels directly determine cellular susceptibility to cuproptosis. In models of cholestatic liver injury and hepatocellular carcinoma resistance, downregulation of FDX1 frequently correlates with cuproptosis resistance. Conversely, restoring FDX1 function or employing copper ion carriers (such as elesclomol) reactivates this lethal pathway [179,180]. Furthermore, FDX1 is regulated by various natural bioactive molecules; for instance, icariin directly binds to and downregulates FDX1, thereby mitigating copper toxicity in neurons [181].

The direct target of copper-mediated cell death is the lipoylated protein within the mitochondrial TCA cycle, specifically the E2 subunit dihydrolipoamide S-acetyltransferase (DLAT) of the pyruvate dehydrogenase complex. The functional activity of DLAT is strictly dependent upon its lipoylation modification at lysine residues [180,182]. Following FDX1-mediated reduction of copper to Cu^+^, excess Cu^+^ specifically binds to the lipoylated lysine residue of DLAT. This aberrant binding directly induces abnormal oligomerisation of the DLAT protein, forming insoluble protein aggregates [175,178]. Multiple studies confirm that DLAT oligomerisation is a hallmark event in cuproptosis: it is observed in nanoplastic-copper co-exposure-induced neurotoxicity and in spinal neurons undergoing I/R injury. This abnormal DLAT oligomerisation not only causes loss of function in the pyruvate dehydrogenase complex, blocking the conversion of pyruvate to acetyl-CoA, but also triggers a cascade of catastrophic events. Most notably, it leads to the destabilisation and loss of iron-sulphur (Fe-S) cluster proteins, including FDX1 itself and the lipoyl synthase (LIAS) [183,184]. This widespread loss of Fe-S cluster proteins results in comprehensive mitochondrial metabolic collapse and severe proteotoxic stress [185]. Notably, the occurrence of cuproptosis correlates closely with cellular metabolic phenotypes: Cells exhibiting high glycolytic activity (such as non-small cell lung carcinoma) typically resist cuproptosis, whereas forcibly redirecting cellular metabolism from glycolysis to the tricarboxylic acid cycle and OXPHOS via metabolic reprogramming pathways like pyruvate kinase M2 (PKM2)/HIF-1α significantly enhances susceptibility to cuproptosis [178,186]. In myocardial I/R injury, silencing FDX1 to block DLAT oligomerisation has been demonstrated to effectively reduce infarct size, further confirming this pathway’s critical role in maintaining homeostasis in energy-intensive organs [187].

#### 3.3.2. Proteotoxic Stress and Mitochondrial Respiratory Failure

The molecular basis of cuproptosis involves mitochondrial metabolic collapse induced by copper overload. As an electron shuttle, FDX1 not only reduces Cu^2+^ to Cu^+^ but, more critically, promotes the lipoylation modification of enzymes in the mitochondrial TCA cycle, such as DLAT [188,189]. However, excess Cu^+^ directly binds these acylated proteins, inducing abnormal aggregation and oligomerisation of DLAT, leading to the formation of insoluble protein aggregates [190,191]. These aggregates not only inactivate DLAT, directly blocking the conversion of pyruvate to acetyl-CoA in the TCA cycle, but also trigger severe proteotoxic stress (Proteotoxic Stress). Concurrently, copper overload destabilises and depletes iron-sulphur cluster (Fe-S cluster) proteins—such as FDX1 itself and FDX2—further exacerbating mitochondrial respiratory chain complex dysfunction [189,192]. This mitochondrial failure, jointly driven by protein aggregation and Fe-S cluster depletion, constitutes a hallmark distinguishing cuproptosis from other forms of cell death [174,175].

Cuproptosis is highly dependent on the mitochondrial respiratory state of the cell. Cells that rely heavily on the TCA cycle and OXPHOS for energy are more susceptible to cuproptosis, whereas cells primarily dependent on glycolysis exhibit a degree of resistance [191,193]. Upon activation of the cuproptosis pathway, TCA cycle inhibition directly disrupts NADH and ATP synthesis, compelling cells to undergo metabolic reprogramming. This involves shifting from efficient OXPHOS to less efficient glycolysis in an attempt to sustain viability [178,190]. However, this metabolic flexibility proves fatally deficient under pathological conditions. In energy-intensive organs such as the heart, mitochondria supply over 70% of ATP; collapse of the TCA cycle signifies not merely energy depletion but the loss of metabolic adaptability [187,194]. Studies indicate that forcing cells to maintain high levels of mitochondrial respiration by inhibiting glycolysis (e.g., using PKM2 activators or lactate dehydrogenase inhibitors) significantly enhances sensitivity to cuproptosis, providing reverse evidence for the critical role of metabolic reprogramming in regulating cuproptosis [178,186].

In the context of cardiovascular disease, mitochondrial respiratory failure triggered by cuproptosis may contribute to the progression from myocardial hypertrophy to heart failure. Owing to the lack of metabolic plasticity in cardiomyocytes, comparable to that observed in tumour cells, the aggregation and inactivation of TCA cycle enzymes such as DLAT directly precipitate a disruption in myocardial energy supply [187,190]. This metabolic dysfunction not only induces acute ATP depletion but also triggers extensive oxidative stress and immunogenic cell death (ICD) via ROS storms and Fe-S cluster protein loss, further exacerbating myocardial inflammation and fibrosis [65,195]. Particularly in myocardial ischaemia-reperfusion injury (MIRI), ROS surges form a vicious cycle with copper overload, accelerating cardiomyocyte death via the FDX1-DLAT axis. Targeted inhibition of FDX1 or clearance of mitochondrial ROS effectively reverses this process and improves cardiac function [187].

#### 3.3.3. The Bidirectional Regulation of Copper and Vascular Remodelling

**Figure 3:**
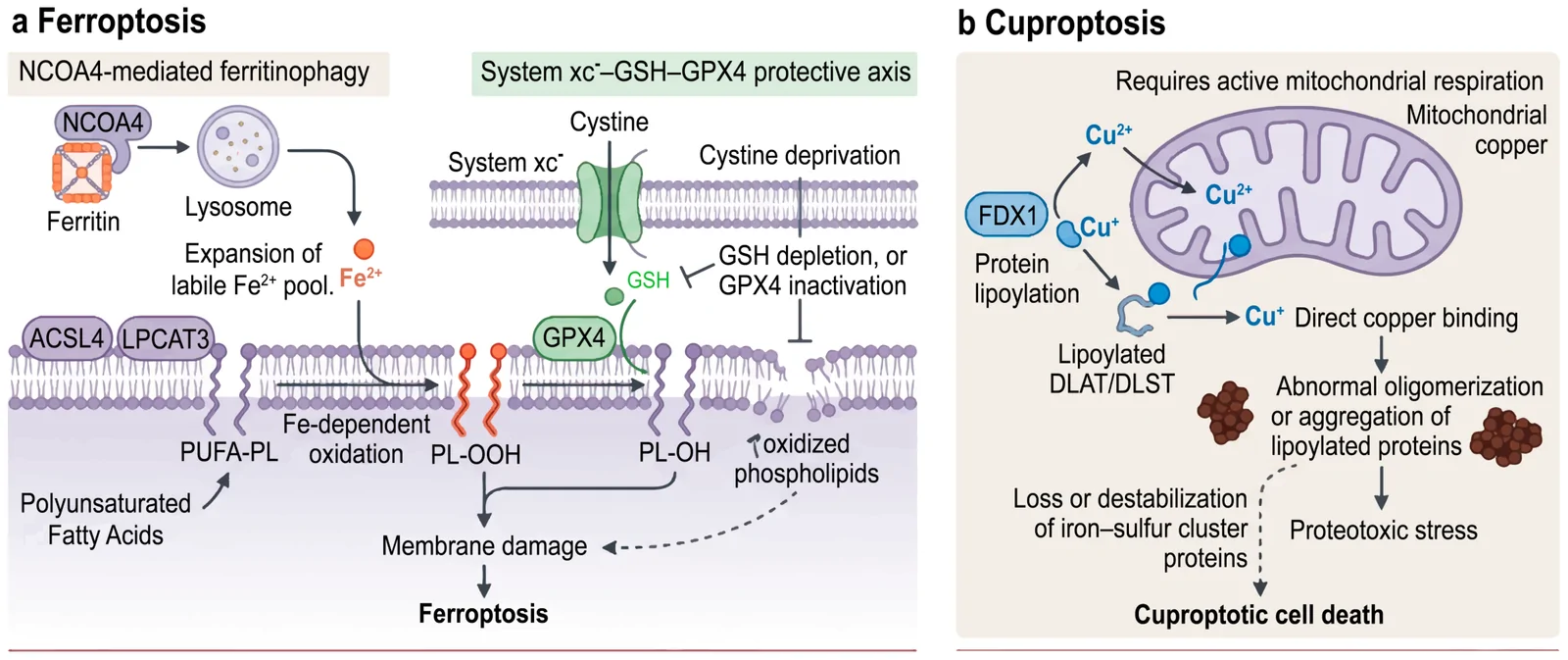
Distinct molecular mechanisms of ferroptosis and cuproptosis. (**a**) During ferroptosis, nuclear receptor coactivator 4 (NCOA4)-mediated ferritinophagy delivers ferritin to lysosomes, releasing iron and expanding the labile Fe^2+^ pool. Acyl-CoA synthetase long-chain family member 4 (ACSL4) and lysophosphatidylcholine acyltransferase 3 (LPCAT3) promote the incorporation of polyunsaturated fatty acids into membrane phospholipids (PUFA-PL), which undergo Fe-dependent oxidation to phospholipid hydroperoxides (PL-OOH). The system xc^−^–glutathione–glutathione peroxidase 4 (system xc^−^–GSH–GPX4) axis normally limits this process by reducing PL-OOH to non-toxic phospholipid alcohols (PL-OH). Cystine deprivation, GSH depletion, or GPX4 inactivation permits PL-OOH accumulation, membrane damage, and ferroptosis. (**b**) In cells dependent on mitochondrial respiration, ferredoxin 1 (FDX1)-associated copper reduction and protein lipoylation facilitate the binding of excess mitochondrial Cu^+^ to lipoylated dihydrolipoamide S-acetyltransferase/dihydrolipoamide S-succinyltransferase (DLAT/DLST). This interaction promotes abnormal oligomerization and aggregation of lipoylated proteins, destabilization or loss of iron–sulfur cluster proteins, proteotoxic stress, and cuproptotic cell death. Solid arrows indicate direct mechanistic steps, whereas dashed arrows denote indirect or integrative mechanistic relationships. Although the core regulated cell-death mechanisms have been demonstrated experimentally, their cardiovascular and cerebrovascular relevance remains predominantly preclinical or associative, particularly for cuproptosis. Original figure created by the authors.

As shown in Figure 3, copper ions at physiological concentrations serve as a key driver of angiogenesis, with their pro-angiogenic effects involving intricate molecular regulatory networks. At the cellular signalling level, the copper transporter 1 (CTR1; SLC31A1) plays a central role as a redox sensor; VEGF stimulation induces oxidation (thiolation) of the cytoplasmic C-terminal cysteine residue (Cys189) in CTR1, facilitating the formation of a disulphide-linked complex between CTR1 and vascular endothelial growth factor receptor 2 (VEGFR2). This complex drives VEGFR2 endocytosis and sustained signal transduction, thereby initiating angiogenesis [196]. Furthermore, the P-type ATPase transporter ATP7A maintains VEGFR2 surface levels and signalling activity by limiting its autophagic degradation. Mechanistically, VEGF promotes ATP7A translocation to the plasma membrane where it binds VEGFR2, preventing association with the autophagic adaptor p62/SQSTM1 and thus averting VEGFR2 entry into the lysosomal degradation pathway [197]. At the transcriptional level, copper ion release significantly upregulates HIF-1α and its downstream target genes VEGF and bFGF, promoting endothelial cell proliferation, migration, and tubulogenesis capabilities [198,199,200]. This pro-angiogenic property has been extensively applied in tissue engineering, such as promoting diabetic wound healing, bone defect repair, and the endothelialisation of small-calibre artificial blood vessels via copper-doped bioactive glass or MOFs [201,202,203].

Although moderate copper promotes vascular repair, under specific pathological conditions, abnormal or excessive copper signalling may inhibit angiogenesis or induce cell death through various mechanisms, demonstrating its bidirectional regulatory properties in vascular remodelling. Research indicates that intervention with carbon quantum dot/Cu_2_O composites significantly downregulates matrix metalloproteinase (MMP-2 and MMP-9) expression and disrupts the F-actin cytoskeleton, thereby inhibiting VEGFR2 expression. This induces cell death in pathological cells (such as ovarian cancer cells) and suppresses their angiogenic capacity [204]. This finding suggests a close association between copper-mediated MMP activation and vascular matrix remodelling. Conversely, within the tumour microenvironment, trapping copper ions using copper deprivators such as TMECN not only blocks PD-L1 transcription and stability but also markedly inhibits tumour neovascularisation, suppressing pathological tissue proliferation via a ‘starvation therapy’ mechanism [205]. This demonstrates that modulating local copper concentrations can switch between promoting physiological repair (such as vascular regeneration following I/R [206]) and suppressing pathological proliferation.

To precisely regulate copper’s bidirectional role in vascular remodelling, achieving ‘on-demand release’ of copper ions via microenvironment-responsive biomaterials has become a key strategy. For instance, MXene-supported copper metal–organic framework (Cu-MOF)-based heterostructures [199] and ZnSrMo-LDH/Cu nanoenzymes [206] intelligently release Cu^2+^ in acidic or inflammatory microenvironments. This simultaneously scavenges ROS while promoting vasodilation and regeneration by upregulating eNOS and NO production [207]. In challenging-to-heal wounds such as diabetic ulcers, this controlled copper release not only exerts antimicrobial effects via photothermal or chemotherapeutic mechanisms but also synergistically modulates macrophage polarisation towards the reparative M2 phenotype, thereby fostering an immunological microenvironment conducive to angiogenesis [199,208]. These microenvironment-responsive strategies are intended to preserve the pro-angiogenic effects of local copper delivery while limiting toxicity from uncontrolled exposure; however, their efficacy and safety remain to be established clinically.

Accordingly, the transition from physiological copper signalling to cuproptotic toxicity depends not on total copper alone, but on mitochondrial copper availability, FDX1-dependent protein lipoylation, and cellular reliance on oxidative phosphorylation.

### 3.4. Zinc Dyshomeostasis: From Oxidative Defence Paralysis to Excitotoxicity

#### 3.4.1. Pathological Reversal of Enzyme Function: eNOS Decoupling and Nitrosative Damage

The homeostasis of zinc ions is crucial for maintaining the redox balance of vascular endothelial cells. Any disruption not only results in the loss of antioxidant defence capabilities but also leads to the pathological reversal of key enzyme functions. Research indicates that zinc deficiency or an imbalance in the zinc-to-copper ratio directly impairs the activity of the NOS system. In zinc-restricted pathological models, serine 1177 phosphorylation levels of eNOS are markedly reduced, leading to diminished NOS activity and impaired NO bioavailability. This alteration constitutes an early molecular basis for cardiovascular disease development [100]. Furthermore, proteins containing zinc finger structures (such as ZFYVE21) are crucial for stabilising eNOS. Disruption of zinc homeostasis leads to reduced expression of these stabilising factors, subsequently inducing functional instability of eNOS and endothelial dysfunction of the glomerular filtration barrier [209]. This zinc-induced decline in enzyme stability not only impedes vasodilator synthesis but also propels endothelial cells towards a pro-inflammatory and pro-apoptotic pathological state [100,210]. 

Under more severe oxidative conditions, eNOS can undergo ‘functional reversal’, whereby it generates ROS rather than NO. In middle-aged rats, increasing dietary copper from 6.45 to 12.9 mg Cu/kg diet, while dietary zinc remained at 15.9 mg Zn/kg diet, increased the serum Cu/Zn molar ratio from 1.018 ± 0.211 to 1.396 ± 0.479 and was accompanied by increased superoxide and H_2_O_2_ in aortic rings [211]. These values are specific to this experimental model and should not be interpreted as universal non-toxic or toxic thresholds. Within this oxidative environment, superoxide anions generated by decoupled eNOS readily undergo diffusion-controlled reactions with residual NO, producing peroxynitrite—a compound exhibiting potent oxidative and cytotoxic properties. Peroxynitrite formation acts as a chemical accelerator of vascular stiffening and fibrosis. It attacks intracellular zinc-sulphur clusters, causing zinc ions to be released from enzymes such as SIRT1. This further inactivates the enzyme and exacerbates abnormal ECM remodelling [212]. This peroxynitrite-mediated nitrosative stress also promotes abnormal accumulation of type IV collagen, leading to thickening of the microvascular basement membrane. Conversely, restoring Cu/Zn-SOD activity aids in scavenging precursor superoxide anions, thereby blocking peroxynitrite generation and its downstream fibrotic cascade reactions [213].

This oxidative and nitrosative damage, triggered by zinc homeostasis imbalance, ultimately leads to severe dysfunction or even death of vascular endothelial cells. Specific zinc-based nanocomposites, when inducing simultaneous bursts of superoxide anion and NO within cells, have been demonstrated to trigger a non-apoptotic form of cell death. This is accompanied by loss of mitochondrial membrane potential and accumulation of autophagosomes, further corroborating the synergistic role of zinc metabolism disruption and nitrosative stress in endothelial toxicity [214]. Environmental exposure to toxic metals or trace element imbalances can impair NO bioavailability and promote oxidative stress in cardiovascular disease contexts [210]. Consequently, disruption of zinc homeostasis represents not only a collapse of the antioxidant defence system but also initiates eNOS decoupling and the generation of peroxynitrite toxicity, thereby contributing to vascular dysfunction.

#### 3.4.2. Impaired Zinc–Keap1–Nrf2 Signalling Under Metabolic Stress

Zinc-dependent regulation of Nrf2 involves defined Keap1 residues rather than Keap1 cysteines collectively. The minimal Zn^2+^-sensing module comprises His225, Cys226, and Cys613; Zn^2+^ coordination induces a conformational change that impairs Cul3–Keap1-mediated Nrf2 ubiquitination [82]. Electrophile sensing can involve other Keap1 cysteines and should be distinguished from this Zn^2+^-binding mechanism. The identification of His225, Cys226, and Cys613 as the minimal zinc-sensing module is supported by direct mechanistic studies.

In experimental diabetic cardiomyopathy, combined sulforaphane and zinc treatment enhanced Nrf2-associated antioxidant responses and reduced oxidative, inflammatory, and fibrotic cardiac injury [215]. Pharmacological Nrf2 activation has also reduced oxidative and inflammatory injury in experimental diabetic nephropathy and retinal-cell models [216,217]. However, direct Zn^2+^ binding to Keap1, indirect pharmacological activation of Nrf2, and correction of systemic zinc deficiency represent distinct mechanisms and should not be treated as equivalent.

#### 3.4.3. Zinc-Associated Mitochondrial Injury in Ischaemic Neurons

In cerebral ischaemia–reperfusion, dysregulated zinc redistribution can contribute to neuronal injury through mitochondrial and redox mechanisms. Research confirms that ischaemia triggers the massive release of zinc stored in a free state within presynaptic vesicles of forebrain glutamatergic neurons (particularly in the hippocampal CA1 region), causing an instantaneous surge in synaptic cleft zinc concentration [218]. Excess zinc ions subsequently flood into postsynaptic neurons, where they are misrecognised and taken up by the mitochondrial calcium uniporter (MCU), leading to persistent pathological accumulation of zinc ions within mitochondria [219,220]. This mitochondrial zinc overload constitutes a progressive toxic event persisting for hours post-ischaemia, directly disrupting mitochondrial structural and functional integrity [221].

**Figure 4:**
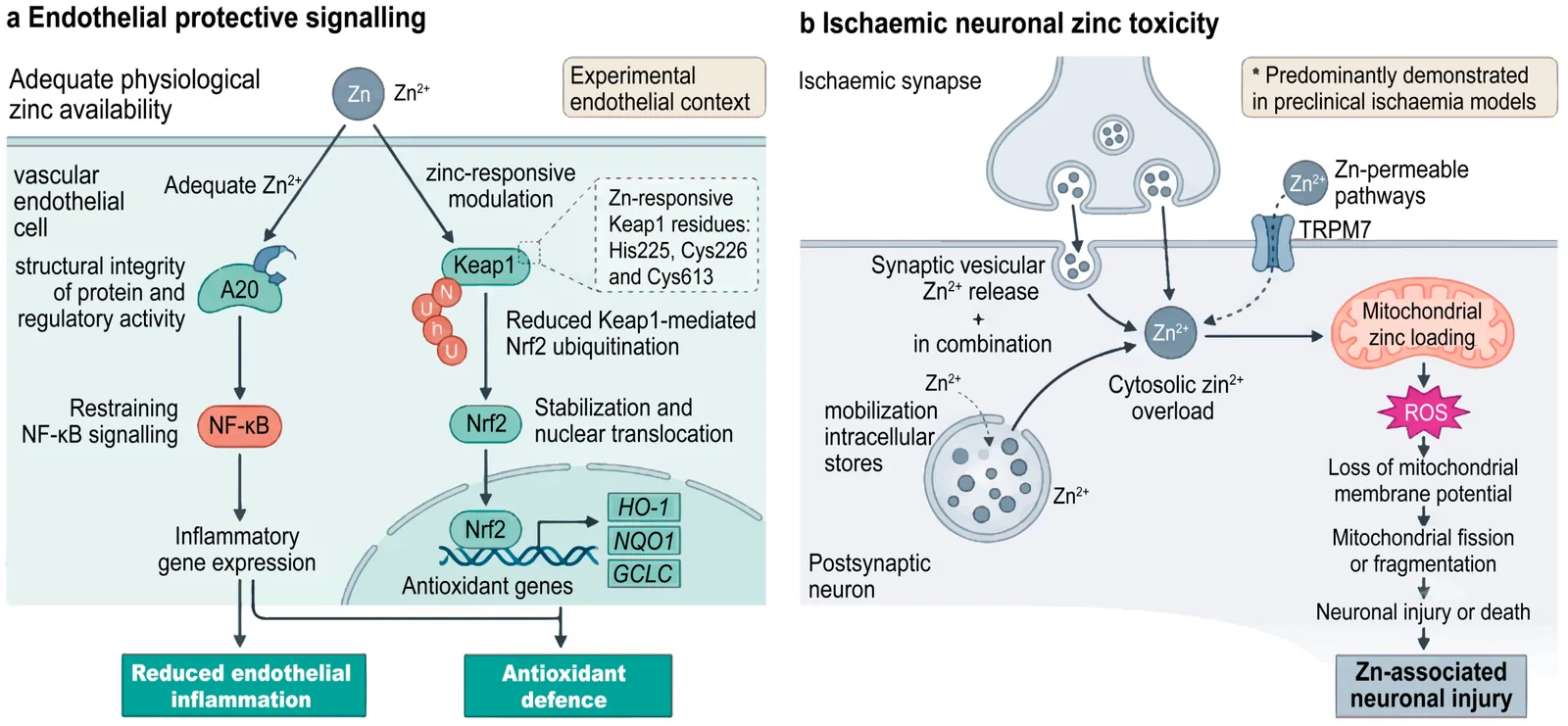
Context-dependent endothelial-protective and neurotoxic effects of zinc. (**a**) Adequate physiological Zn^2+^ availability supports the structural integrity and regulatory activity of the zinc-finger protein tumour necrosis factor alpha-induced protein 3 (A20), thereby restraining nuclear factor-κB (NF-κB) signalling and endothelial inflammatory gene expression. Zinc-responsive modulation of Kelch-like ECH-associated protein 1 (Keap1), including experimentally implicated His225, Cys226, and Cys613 residues, reduces Keap1-mediated ubiquitination of nuclear factor erythroid 2-related factor 2 (Nrf2). Stabilized Nrf2 translocates to the nucleus and induces antioxidant genes, including haem oxygenase 1 (HO-1), NAD(P)H quinone dehydrogenase 1 (NQO1), and glutamate–cysteine ligase catalytic subunit (GCLC), supporting antioxidant defence and reduced endothelial inflammation. (**b**) During cerebral ischaemia, synaptic Zn^2+^ release and Zn^2+^ mobilization from intracellular stores contribute to cytosolic zinc overload. Zn-permeable pathways, potentially including transient receptor potential melastatin 7 (TRPM7), may facilitate zinc entry or redistribution. Mitochondrial zinc loading promotes reactive oxygen species (ROS) production, loss of mitochondrial membrane potential, mitochondrial fission or fragmentation, and zinc-associated neuronal injury. Dashed arrows indicate context-dependent or incompletely resolved routes. The neurotoxic pathway has been demonstrated predominantly in preclinical models of cerebral ischaemia. Original figure created by the authors.

As shown in Figure 4, mitochondrial zinc accumulation has been implicated in mitochondrial dysfunction and oxidative injury in experimental ischaemia models. Research indicates that zinc accumulation within mitochondria directly induces pathological depolarisation, swelling, and fragmentation of mitochondria, a process highly dependent on the dynamin-related protein 1 (Drp1)-mediated mitochondrial fission mechanism [219,222]. More critically, zinc ion accumulation serves as an upstream initiator of the early ROS burst during I/R. During the reperfusion phase (approximately 40 min post-ischaemia), transient mitochondrial hyperpolarisation synergises with persistent zinc accumulation to trigger a second, intense wave of ROS surges [220]. Moreover, zinc toxicity involves temporally and spatially amplified positive feedback loops: ROS generated by mitochondria further activate NADPH oxidase, leading to subsequent ROS production and more severe intracellular zinc accumulation. This forms a vicious ‘zinc-ROS’ cycle that accelerates neuronal death [9].

The destructive effects of zinc toxicity extend beyond neurons to involve vascular endothelium and glial cells, forming a pathological network throughout the brain. Within cerebral microvascular endothelial cells, ischaemia-induced mitochondrial zinc overload activates MMP-2, directly causing degradation of tight junction proteins and increased blood–brain barrier (BBB) permeability, thereby exacerbating vasogenic oedema [222]. Concurrently, high concentrations of zinc ions released from ischaemic neurons into the extracellular space inhibit glutamate uptake by astrocytes, intensifying excitotoxicity. Acting as a “sensitising agent” on microglia, zinc promotes their polarisation towards a pro-inflammatory M1 phenotype, triggering the release of numerous pro-inflammatory mediators that further deteriorate the microenvironment of the ischaemic penumbra [218].

## 4. Disease-Specific Manifestations of Metal Dyshomeostasis and Oxidative Stress

### 4.1. Atherosclerosis

#### 4.1.1. Fe: Intraplaque Haemorrhage and Macrophage Ferroptosis

In advanced atherosclerotic plaques, fragile neovessels and endothelial disruption permit intraplaque haemorrhage, introducing erythrocytes and haemoglobin-derived iron into the intima [223,224]. Haemorrhagic regions of human carotid plaques consequently show marked iron deposition together with altered lipid and glutamate/glutamine metabolism [224]. However, histological iron deposition indicates an increased local iron burden but does not directly quantify the redox-active labile iron pool. Haemodynamic injury may also permit erythrocyte entry into the intima during earlier stages of lesion development [223].

Erythrophagocytosis further increases the iron burden of plaque macrophages. Acquisition of an M(heme) phenotype may facilitate haem processing and iron recycling [225], whereas persistent iron loading increases susceptibility to macrophage ferroptosis [11]. Ferroptotic and iron-loaded macrophages promote inflammatory signalling and matrix metalloproteinase release, thereby contributing to fibrous-cap weakening [226]. Within CD163-positive macrophage populations, iron-associated NF-κB activation has also been linked to endothelial-to-mesenchymal transition and plaque instability [226]. P2Y12-dependent restriction of macrophage iron efflux may further promote iron retention and necrotic-core formation [227]. The general redox chemistry and ferroptotic mechanisms underlying these effects are described in Section 3.1 and Section 3.2.

#### 4.1.2. Cu: Copper-Catalysed Low-Density Lipoprotein Oxidation and Foam-Cell Formation

Copper readily catalyses low-density lipoprotein (LDL) oxidation in experimental systems, generating lipid hydroperoxides, malondialdehyde, and pro-inflammatory oxylipins [228,229,230]. Atomic-force microscopy further showed that copper-oxidised LDL particles exhibit altered size, stiffness, and adhesiveness, properties that may favour their retention within the subendothelial space [228].

Oxidised LDL is recognised and internalised by endothelial cells and macrophages through lectin-like oxidised LDL receptor-1, promoting intracellular lipid accumulation and inflammatory signalling [231,232,233]. Oxidised LDL-containing immune complexes can further enhance macrophage proliferation and responses associated with plaque vulnerability [234]. Copper oxide nanoparticle exposure has also been shown to disrupt macrophage lysosomal function and cholesterol efflux, thereby promoting foam-cell formation [235]. Because this evidence derives from an exogenous nanoparticle model, it should not be equated directly with endogenous copper accumulation in atherosclerotic plaques. Copper-dependent redox chemistry is discussed in Section 3.1.

#### 4.1.3. Zn: Context-Dependent Modulation of Endothelial Inflammation and Matrix Turnover

Evidence for a uniformly plaque-stabilising effect of zinc remains limited. In endothelial cells exposed to oxidised LDL, activation of the zinc-sensing receptor GPR39 with the synthetic agonist TC-G 1008 reduced monocyte adhesion and the expression of vascular cell adhesion molecule-1 and E-selectin, consistent with reduced NF-κB signalling [236]. Because this intervention involved pharmacological receptor activation rather than zinc supplementation, it does not establish that increasing systemic or intracellular zinc concentrations stabilises atherosclerotic plaques.

**Figure 5:**
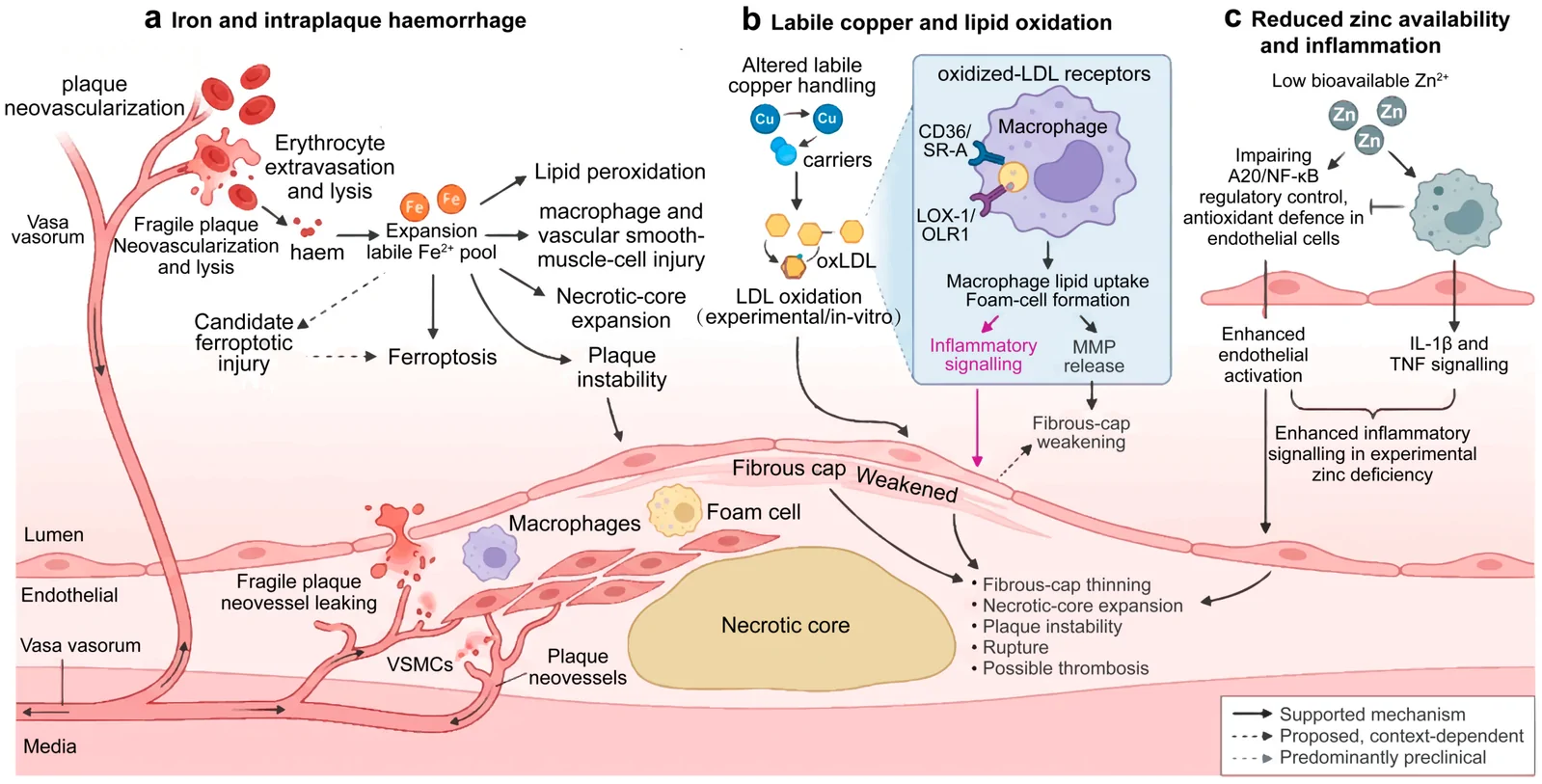
Potential contributions of iron, copper, and zinc dyshomeostasis to atherosclerotic plaque instability. (**a**) Plaque neovascularization and leakage from fragile neovessels promote erythrocyte extravasation and lysis, haem release, and expansion of the labile Fe^2+^ pool. Iron-dependent lipid peroxidation may contribute to macrophage and vascular smooth-muscle-cell injury, necrotic-core expansion, and candidate ferroptotic injury, thereby favouring plaque instability. (**b**) Altered labile copper handling may promote low-density lipoprotein (LDL) oxidation under experimental conditions. Oxidized LDL (ox-LDL) is recognized by scavenger receptors, including cluster of differentiation 36 (CD36), scavenger receptor A (SR-A), and lectin-like oxidized LDL receptor 1 (LOX-1/OLR1), promoting macrophage lipid uptake, foam-cell formation, inflammatory signalling, matrix metalloproteinase (MMP) release, and fibrous-cap weakening. (**c**) Reduced zinc availability may impair A20/NF-κB regulatory control and antioxidant defence in endothelial cells and macrophages, enhancing endothelial activation and interleukin-1β (IL-1β) and tumour necrosis factor (TNF) signalling. These metal-dependent pathways may converge on fibrous-cap thinning, necrotic-core expansion, plaque instability, rupture, and thrombosis. Solid arrows indicate supported mechanisms; dark dashed arrows indicate proposed or context-dependent relationships; light dashed arrows indicate predominantly preclinical evidence. A20, tumour necrosis factor alpha-induced protein 3; NF-κB, nuclear factor kappa B; VSMCs, vascular smooth muscle cells. Original figure created by the authors.

Zinc is also incorporated into the catalytic centres of matrix metalloproteinases, including MMP-9, which participate in extracellular-matrix turnover and plaque instability [237]. However, the structural requirement for zinc within an enzyme does not demonstrate that zinc supplementation will reproduce or enhance the enzyme’s pathological or protective effects. Current evidence therefore supports a context-dependent relationship between zinc-associated signalling, endothelial inflammation, and matrix remodelling rather than a uniformly protective role. These metal-associated pathways are summarized in Figure 5.

### 4.2. Hypertension and Endothelial Dysfunction

#### 4.2.1. Fe: Angiotensin II–Associated Iron Loading and Vascular Remodelling

In experimental Ang II-driven hypertension, activation of vascular NADPH oxidases is accompanied by altered iron handling and increased oxidative stress [238,239,240,241]. Iron loading can enhance superoxide-associated loss of NO bioavailability and promote inflammatory and fibrotic signalling within the vascular wall. These findings derive predominantly from Ang II stimulation and experimental iron-loading models and do not demonstrate that systemic iron excess occurs in all forms of human hypertension.

A more direct link between Ang II signalling and cellular iron uptake is provided by TfR1. Ang II increases TfR1 expression in VSMCs, thereby promoting intracellular iron accumulation, proliferation, and migration. Genetic deletion or antibody-mediated inhibition of TfR1 attenuates intima–media thickening and vascular remodelling [242,243]. In vascular smooth muscle and renal tubular cells, these changes are accompanied by GPX4 loss, lipid-peroxide accumulation, and mitochondrial dysfunction [242,244].

The Elabela–apelin receptor (APJ) axis has been reported to reduce Ang II-associated ferroptotic signalling through Nrf2/GPX4 activation, with corresponding reductions in experimental myocardial remodelling and fibrosis [245]. This intervention supports the involvement of iron-dependent lipid peroxidation in hypertensive organ injury but remains preclinical. The underlying Fenton chemistry and ferroptotic pathways are described in Section 3.1 and Section 3.2.

#### 4.2.2. Copper Homeostasis, Cu/Zn-SOD Activity, and Vascular Reactivity

Copper is required for the maturation and catalytic activity of Cu/Zn-superoxide dismutase, including extracellular SOD within the vascular wall [246,247]. By limiting superoxide accumulation, this enzyme helps preserve NO bioavailability and endothelium-dependent vasodilation.

Berberine, finerenone, and resveratrol have been associated with restoration of Cu/Zn-SOD expression or activity, reduced superoxide accumulation, and improved NO signalling in experimental vascular models [247,248,249]. These studies support the importance of the Cu/Zn-SOD system but do not evaluate copper supplementation itself. Pharmacological regulation of SOD expression should therefore not be interpreted as direct evidence that increasing copper intake improves endothelial function.

Conversely, excessive redox-active copper can increase oxidative stress and alter vascular responses to acetylcholine and noradrenaline in experimental models [211,250]. ATP7A contributes to vascular copper homeostasis by supporting copper delivery within the secretory pathway and cellular copper efflux [251]. Copper effects on vascular tone therefore depend on copper availability and intracellular handling rather than being uniformly protective or harmful. The physiological regulation of Cu/Zn-SOD and copper-dependent redox chemistry is described in Section 2.2.1 and Section 3.1.

#### 4.2.3. Zn: Zinc-Dependent eNOS Stability and NO Bioavailability

Zinc contributes to the structural stability of the eNOS homodimer through a zinc–thiolate centre formed by cysteine residues at the subunit interface [252,253]. Integrity of this centre supports the dimeric conformation required for coupled electron transfer and NO production.

Under zinc-restricted or strongly oxidative conditions, disruption of the zinc–thiolate centre can destabilise the eNOS dimer and favour enzymatic uncoupling [252,254]. Uncoupled eNOS generates superoxide rather than NO, thereby reducing NO bioavailability and promoting peroxynitrite formation. These changes impair endothelium-dependent vasodilation and may contribute to elevated vascular tone. Zinc loss is nevertheless one of several redox and cofactor abnormalities capable of promoting eNOS uncoupling.

Prenatal and postnatal zinc restriction produced persistent reductions in eNOS expression and activity and increased systolic blood pressure in male rat offspring [255]. The incomplete reversal observed after adult zinc supplementation indicates that developmental timing influenced the phenotype in this model; it does not establish that zinc supplementation reverses established adult hypertension. Cadmium and lead may also disrupt the zinc-dependent eNOS structure through metal displacement [256], although toxicant-induced displacement and nutritional zinc deficiency represent different exposure conditions. The downstream chemistry of eNOS uncoupling is described in Section 3.4.1.

### 4.3. Ischaemic Heart Disease and Myocardial Ischaemia–Reperfusion Injury

#### 4.3.1. Fe: Reperfusion-Associated Iron Dysregulation and Ferroptotic Injury

During myocardial ischaemia–reperfusion, disruption of iron handling intersects with the restoration of oxygen supply and mitochondrial electron flow. Myocardial proteomic evidence has demonstrated GPX4 downregulation during infarction [117]. During reperfusion, electron leakage from damaged mitochondria increases ROS generation, allowing redox-active iron to amplify oxidative injury through Fenton chemistry and membrane lipid peroxidation.

Reduced GPX4 activity limits the detoxification of phospholipid hydroperoxides and increases cardiomyocyte susceptibility to ferroptosis [18,117]. Iron-dependent lipid peroxidation can further impair mitochondrial membrane integrity, membrane potential, and ATP production [18]. The cited myocardial studies support an important contribution of ferroptotic injury but do not quantify its relative contribution compared with other forms of cardiomyocyte damage during reperfusion.

**Figure 6:**
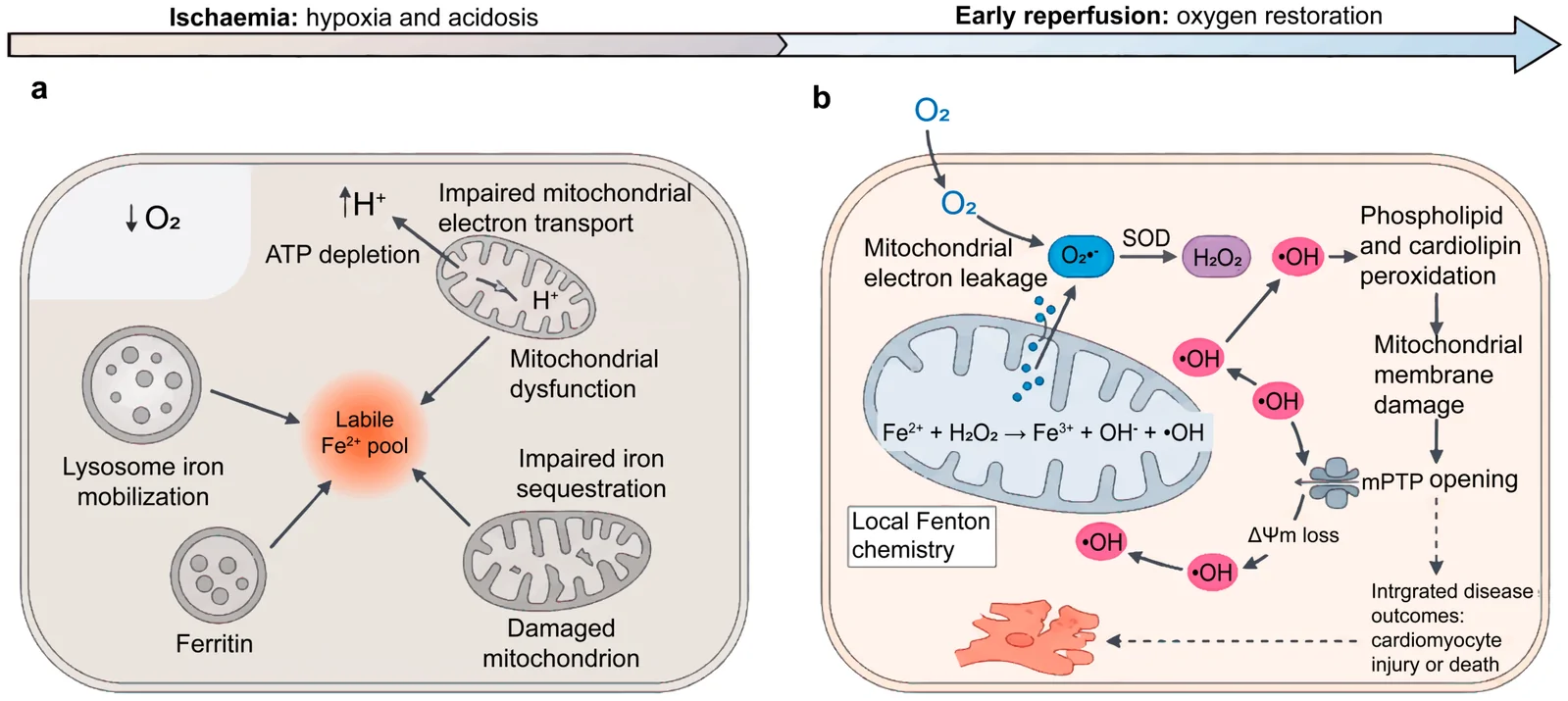
Iron-dependent oxidative injury during myocardial ischaemia–reperfusion. (**a**) During myocardial ischaemia, reduced oxygen availability, intracellular acidosis, adenosine triphosphate (ATP) depletion, and impaired mitochondrial electron transport contribute to mitochondrial dysfunction. Lysosomal or ferritin-associated iron mobilization, impaired iron sequestration, and disturbed mitochondrial iron handling expand the labile Fe^2+^ pool. (**b**) During early reperfusion, oxygen restoration promotes mitochondrial electron leakage and superoxide (O_2_•^−^) production. Superoxide dismutase (SOD) converts O_2_•^−^ to H_2_O_2_, which reacts locally with Fe^2+^ through Fenton chemistry to generate •OH. Local hydroxyl-radical production promotes cardiolipin and phospholipid peroxidation, mitochondrial membrane damage, mitochondrial permeability transition pore (mPTP) opening, loss of mitochondrial membrane potential (ΔΨm), and cardiomyocyte injury or death. Solid arrows indicate direct biochemical or cellular events, whereas dashed arrows indicate integrated disease outcomes. The depicted mechanisms are supported predominantly by experimental models of myocardial ischaemia–reperfusion. Original figure created by the authors.

Preclinical interventions that preserve mitochondrial function or limit lipid peroxidation provide additional mechanistic support. Cardiosphere-derived extracellular vesicles carrying ATP5a1 improved mitochondrial function and reduced experimental myocardial ischaemia–reperfusion injury [257]. These findings remain preclinical, and detailed therapeutic evaluation is provided in Section 5. The general mechanisms of Fenton chemistry and ferroptosis are described in Section 3.1 and Section 3.2. The iron-dependent events during myocardial ischaemia–reperfusion are summarized in Figure 6.

#### 4.3.2. Cu: Bidirectional Effects of Mitochondrial Copper Imbalance

Cardiomyocytes require copper for the activity of cytochrome c oxidase and efficient mitochondrial respiration [258]. Chronic copper deficiency reduces cytochrome c oxidase activity, impairs OXPHOS, and increases reliance on glycolytic metabolism. These metabolic changes can contribute to energetic stress, myocardial hypertrophy, and ventricular dysfunction. Chronic nutritional deficiency should nevertheless be distinguished from acute copper redistribution during myocardial ischaemia–reperfusion.

Experimental myocardial ischaemia–reperfusion studies more directly implicate impaired copper efflux and mitochondrial copper accumulation. Sleep fragmentation increased sympathetic and noradrenergic signalling, inhibited ATP7A-mediated copper efflux, and aggravated myocardial copper accumulation and ischaemia–reperfusion injury [259]. Excess mitochondrial copper can promote cuproptosis through aggregation of lipoylated mitochondrial proteins and destabilisation of iron–sulphur cluster proteins. In experimental myocardial ischaemia–reperfusion, the PCSK9–LIAS interaction was linked to cardiomyocyte cuproptosis, whereas inhibition of this interaction reduced myocardial injury and adverse remodelling [260].

SIRT3 has also been reported to modulate cardiomyocyte susceptibility to copper accumulation and cuproptosis in pressure-overload models [261]. Pressure-overload remodelling and acute myocardial ischaemia–reperfusion are distinct pathological settings, although both support a relationship between mitochondrial copper handling and cardiac injury. The detailed molecular execution of cuproptosis is described in Section 3.3.

#### 4.3.3. Zn: Zinc Transport and Post-Ischaemic Cardiac Repair

Direct cardiac evidence linking dynamic zinc redistribution to myocardial ischaemia–reperfusion remains more limited than that for iron. In mouse hearts, downregulation of the zinc transporter ZIP13 impaired mitochondrial iron–sulphur cluster biosynthesis, increased ferroptotic susceptibility, and aggravated ischaemia–reperfusion injury [262]. This result links intracellular zinc transport to mitochondrial iron metabolism but does not demonstrate that systemic zinc supplementation during reperfusion is cardioprotective.

Zinc-binding transcriptional proteins may also participate in post-ischaemic repair. ZNF667 promoted angiogenesis after myocardial ischaemia by repressing the anti-angiogenic factor VASH1 and activating Wnt signalling [263]. However, this study evaluated the function of a zinc-finger transcription factor rather than changes in labile Zn^2+^ or zinc supplementation. Zinc-transporter function, labile-zinc signalling, and zinc-finger proteins should therefore not be treated as interchangeable mechanisms.

### 4.4. Ischaemic Stroke

#### 4.4.1. Iron Dysregulation in the Ischaemic Penumbra and Glia–Neuron Crosstalk

Neurons and glial cells within the ischaemic penumbra undergo evolving disturbances in iron uptake, mitochondrial function, and lipid metabolism. Experimental studies have demonstrated iron accumulation and lipid peroxidation in peri-infarct tissue [264,265]. Changes in TfR1 and divalent metal transporter 1 expression increase cellular iron uptake [265,266,267]. Ischaemia-associated lactate accumulation also promotes histone H4 lysine 12 lactylation, increasing *ZIP14* transcription and linking metabolic stress to iron entry and ferroptotic susceptibility [268].

Neuronal vulnerability is further influenced by phospholipid remodelling and mitochondrial dynamics. ACSL4 promotes the incorporation of oxidation-prone polyunsaturated fatty acids into membrane phospholipids and contributes to ferroptotic brain injury after cerebral ischaemia [269]. The ARRDC3–Drp1 axis promotes mitochondrial fission, whereas altered sideroflexin 1 expression links mitochondrial transport and metabolic dysfunction to neuronal survival [270,271]. These processes reinforce iron-dependent lipid peroxidation but do not exclude the simultaneous contribution of excitotoxic, inflammatory, and other injury pathways.

Microglia are both targets and amplifiers of iron-dependent injury. Ferroptotic or metabolically stressed microglia enhance neuroinflammatory signalling through pathways involving NF-κB and cyclic GMP–AMP synthase–stimulator of interferon genes [272,273,274,275]. Foam-cell-derived exosomal miRNA Novel-3 has also been reported to promote microglial ferroptosis and inflammatory injury after stroke [276]. Conversely, heat-shock protein beta-1 and ferritin light chain reduce microglial lipid peroxidation, iron accumulation, or inflammatory signalling [277,278]. The molecular execution of ferroptosis is described in Section 3.2.

#### 4.4.2. Cu: Copper Homeostasis in Post-Ischaemic Vascular Repair

Copper is required for proteins involved in mitochondrial respiration, extracellular-matrix regulation, and angiogenic signalling. In vitro studies using copper-binding peptides or copper complexes indicate that copper availability can influence VEGF, brain-derived neurotrophic factor, and receptor tyrosine kinase signalling [279,280,281,282]. Much of this evidence derives from cell systems or non-stroke repair models and does not establish that copper supplementation improves cerebral revascularisation after acute ischaemic stroke.

More disease-specific evidence is available from chronic cerebral ischaemia. Metallothionein 2A promoted angiogenesis by limiting mitochondrial copper accumulation and preserving mitochondrial function [283]. Excess copper was associated with increased dihydrolipoamide S-acetyltransferase oligomerisation and reduced succinate dehydrogenase iron–sulphur subunit expression, consistent with cuproptosis-associated mitochondrial stress and impaired endothelial repair [283]. Because this model concerns chronic cerebral hypoperfusion, its findings should not be interpreted as direct evidence that cuproptosis dominates acute neuronal death after arterial occlusion.

Ageing further alters post-ischaemic vascular recovery. Reduced hypoxia-inducible factor-1 transcriptional activity has been associated with impaired angiogenesis after cerebral ischaemia in aged animals [284]. COMMD1-related regulation has been proposed as one contributor to this response [284,285], although direct evidence in human stroke remains limited. These studies indicate that both insufficient copper-dependent protein function and excessive mitochondrial copper can impair vascular repair under different experimental conditions.

#### 4.4.3. Zn: Synaptic Zinc Redistribution and Time-Dependent Neurovascular Effects

Zinc is stored in presynaptic vesicles or bound to intracellular proteins, while a labile Zn^2+^ pool participates in neuronal signalling. During cerebral ischaemia, vesicular and protein-bound zinc can be mobilised into the synaptic cleft and cytosol, producing marked redistribution of labile zinc [286,287]. Postsynaptic zinc accumulation is facilitated by voltage-gated calcium channels, transient receptor potential channels, and altered ion-exchange mechanisms. Inhibition of sodium–hydrogen exchanger 1 with amiloride reduced hippocampal zinc accumulation and neuronal death after ischaemia [288]. Carvacrol-mediated inhibition of transient receptor potential melastatin 7 and N-acetyl-L-cysteine-mediated inhibition of transient receptor potential melastatin 2 produced similar protective effects in global cerebral ischaemia models [289,290].

Excitatory amino acid carrier 1 contributes to neuronal cysteine uptake and glutathione synthesis, and its circadian variation influences post-ischaemic hippocampal zinc accumulation [291,292]. Once cytosolic labile zinc rises, mitochondrial uptake can promote Drp1-dependent mitochondrial fission and blood–brain barrier disruption [222]. Mitochondrial ROSs facilitate zinc release during early injury, whereas accumulated zinc subsequently increases NADPH oxidase-dependent oxidant generation [9]. Zinc accumulation has also been linked to cyclin-dependent kinase 5 activation, endoplasmic-reticulum stress, and inflammasome-associated inflammation [293,294,295,296].

Zinc effects after stroke differ between the acute injury and recovery phases. Acute experimental chelation reduced zinc-associated inflammation and endoplasmic-reticulum stress [295,296], whereas zinc administration during the recovery phase promoted astrocyte-dependent hypoxia-inducible factor-1α/VEGF signalling, angiogenesis, and neurological recovery in an experimental stroke model [297]. These findings support a time-dependent relationship but do not define a clinically safe zinc concentration or treatment window. Chelation and supplementation strategies are evaluated separately in Section 5.

### 4.5. Haemorrhagic Stroke

#### 4.5.1. Fe: Haemoglobin-Derived Iron and Secondary Brain Injury

Following intracerebral haemorrhage (ICH), erythrolysis within the haematoma releases haemoglobin into the brain parenchyma, where it is degraded to haem and redox-active iron [298,299,300]. Complement activation, including C3-associated processes, accelerates early erythrolysis, whereas experimental complement inhibition reduces subsequent iron deposition and brain injury [301]. Haem and its oxidised derivatives can perturb cellular structures, activate glial cells, disrupt the blood–brain barrier, and promote inflammation [302,303]. Hemin and free iron have also been linked to nuclear and mitochondrial DNA damage [304,305]. In one experimental study, hemin-induced transient senescence was interpreted as a protective response that limited ferroptosis [304]. In microglia, upregulation of the mitochondrial iron transporter SLC25A28 promotes mitochondrial iron accumulation and glycolytic reprogramming, linking haematoma-derived iron to neuroinflammatory activation [306].

Perihaematomal iron remains elevated after ICH, and greater local iron burden has been associated with larger cerebral oedema volumes [307]. Ferrous iron and ROS amplify lipid peroxidation and ferroptotic signalling, with hypoxia-inducible factor-1α implicated in the acute oxidative and inflammatory response [298,308,309]. Iron-dependent ferroptosis therefore contributes to secondary brain injury together with blood–brain barrier disruption, inflammation, and other forms of cellular damage. The underlying Fenton chemistry and ferroptotic pathways are described in Section 3.1 and Section 3.2.

The duration of exposure to haemoglobin-derived products is also determined by haematoma clearance. Microglia and macrophages remove erythrocytes and haemoglobin through CD36-mediated phagocytosis and the CD163–haem oxygenase-1 pathway [299,310], with CD163 and haem oxygenase-1 expression peaking between days 8 and 15 after ICH [311]. Neutrophil-derived lactoferrin binds iron and facilitates detoxification [312], whereas aquaporin-4-dependent glymphatic and meningeal lymphatic drainage promotes haemoglobin and iron efflux [313]. Toll-like receptor 7 activation has also been reported to enhance haemoglobin clearance through a Bruton’s tyrosine kinase–calreticulin–low-density lipoprotein receptor-related protein 1–haemopexin pathway [314].

#### 4.5.2. Cu: Ceruloplasmin-Dependent Iron Handling After ICH

Ceruloplasmin is a copper-containing ferroxidase that oxidises ferrous iron to ferric iron, thereby facilitating iron binding and transport and limiting the availability of redox-active iron. In experimental haemorrhagic models, ceruloplasmin expression in parenchymal neurons and astrocytes peaks approximately three days after bleeding, and endogenous upregulation or exogenous ceruloplasmin administration reduces blood–brain barrier disruption, cerebral oedema, and neurological deficits [315]. Single-cell transcriptomic analysis of neonatal ICH also identified ceruloplasmin-and haem oxygenase-1-related iron-handling programmes in microglia and macrophages during neurovascular repair [316]. Neonatal repair responses, however, should not be assumed to reproduce those of adult hypertensive ICH.

Clinical observations suggest that ceruloplasmin status may reflect altered copper–iron coupling after ICH. In patients with hypertensive ICH, lower serum ceruloplasmin accompanied by higher small-molecule copper was associated with increased in-hospital mortality and poorer outcome [317]. This association does not establish causality or define copper concentrations within injured brain tissue. Glycosylated ceruloplasmin after cardiac arrest [318], copper-associated cerebral iron deposition in Wilson disease [319], and changes in ceruloplasmin under chronic hypoxic conditions [320,321,322] provide additional evidence for copper–iron interactions but represent distinct disease and sampling contexts. Serum ceruloplasmin, glycosylated ceruloplasmin, small-molecule copper, and cerebrospinal-fluid ceruloplasmin should therefore not be treated as interchangeable measurements.

### 4.6. Abdominal Aortic Aneurysm

#### 4.6.1. Fe: Iron Accumulation, Macrophage Ferroptosis, and Matrix Degradation

Iron deposition has been detected in macrophage-rich regions of the abdominal aortic aneurysm wall, with haemoglobin and haem metabolism proposed as important local iron sources [91,323,324]. In experimental comparisons, saccular aneurysms showed greater iron release, oxidative-product accumulation, and MMP-9 expression than fusiform aneurysms [325]. Molecular magnetic-resonance imaging further demonstrated spatial overlap between inflammatory iron-oxide signals and collagen or elastin degradation, while their combined assessment improved rupture-risk prediction in experimental models [326,327]. These findings associate local iron burden with inflammation and extracellular-matrix damage, although imaging colocalisation does not establish iron deposition as the initiating cause of aneurysm formation.

Macrophages provide a cellular link between iron accumulation and matrix degradation. Single-cell sequencing identified altered expression of ferroptosis-associated genes in abdominal aortic aneurysm macrophages [328]. Activated T lymphocytes can further promote macrophage lipid peroxidation and migration through extracellular vesicles containing PKM2 [329]. Pharmacological inhibition of ferroptosis with Liproxstatin-1 or increased GPX4 expression reduced macrophage infiltration, MMP-2 and MMP-9 expression, and experimental aneurysm formation or enlargement [328,330]. These findings support macrophage ferroptosis as a contributor to inflammatory matrix degradation.

The CD163–haem oxygenase-1 pathway shows contrasting effects in different cellular contexts. CD163-mediated haemoglobin uptake and haem oxygenase-1-mediated haem degradation participate in haem detoxification [91,331]. Increased or stabilised haem oxygenase-1 in VSMCs reduced oxidative stress, apoptosis, MMP expression, and experimental aneurysm progression [332,333,334]. In contrast, macrophage-derived haem oxygenase-1 increased inducible NO synthase-dependent peroxynitrite formation, VSMC apoptosis, and aneurysm development in another experimental model [335]. Thus, the reported effects of haem oxygenase-1 differ according to the cellular context.

#### 4.6.2. Cu: The ATP7A–Copper–LOX Axis in Aortic Wall Cross-Linking

LOX is a copper-dependent amine oxidase that catalyses collagen and elastin cross-linking and contributes to the mechanical stability of the aortic ECM. LOX maturation requires correct intracellular folding and trafficking through the secretory pathway. The p.M292R mutation causes intracellular retention of LOX in the endoplasmic reticulum and prevents its normal transport to the Golgi apparatus. Loss of functional LOX increased susceptibility to aortic dilatation under haemodynamic stress [68]. The documented abnormality is defective intracellular trafficking rather than a demonstrated primary failure of copper binding.

Copper delivery to LOX is regulated by the copper chaperone Atox1 and ATP7A within the secretory pathway. Reduced ATP7A expression or function in VSMCs decreases copper delivery and LOX activity and promotes experimental abdominal aortic aneurysm development [66]. Copper chelation similarly reduces LOX activity and increases aneurysm formation, whereas ATP7A overexpression preserves LOX activity and limits aneurysm development [66]. ATP7A loss is also associated with increased microRNA-125b expression, further suppressing LOX activity and promoting vascular inflammation and matrix degradation.

LOX-mediated cross-linking is important because collagen accumulation without adequate cross-link formation does not provide equivalent resistance to aneurysmal expansion [336]. Excessive prostaglandin E2 receptor EP4 signalling in VSMCs reduces LOX expression, impairs collagen and elastin cross-linking, and promotes experimental aneurysm progression [337]. These findings support impairment of the ATP7A–copper–LOX axis as a contributor to mechanical weakening of the aneurysmal wall [338].

#### 4.6.3. Transient Receptor Potential Melastatin 7–Metal-Regulatory Transcription Factor 1–MMP Signalling and Matrix Degradation

Matrix metalloproteinases are zinc-dependent endopeptidases that participate in extracellular-matrix degradation during abdominal aortic aneurysm development [339]. In addition to the structural requirement for zinc within their catalytic sites, intracellular zinc signalling regulates MMP expression. In VSMCs, the transient receptor potential melastatin 7 (TRPM7) channel kinase promotes Zn^2+^ entry and activates metal-regulatory transcription factor 1 (MTF1), thereby increasing *Mmp2* transcription [340]. Smooth-muscle-cell-specific deletion or pharmacological inhibition of transient receptor potential melastatin 7 reduced this signalling response and suppressed experimental aneurysm formation [340].

Clinical and experimental observations have associated diabetes with lower circulating zinc, reduced MMP-2 activity, and lower aneurysm susceptibility [341]. Dietary zinc supplementation in diabetic mice restored MMP activity and increased aneurysm progression towards that observed in non-diabetic animals [341]. These results support a role for zinc availability in MMP-dependent matrix remodelling in this model. They do not establish that reduced circulating zinc is the sole explanation for the association between diabetes and lower abdominal aortic aneurysm risk or that systemic zinc restriction would be a safe preventive strategy.

### 4.7. Diabetic Angiopathy 

#### 4.7.1. Fe: Hyperglycaemia-Associated Endothelial Ferroptosis and Metabolic Inflammation

Hyperglycaemia increases endothelial ferroptotic susceptibility through coordinated changes in iron uptake, phospholipid remodelling, and antioxidant defence. In type 2 diabetic cardiomyopathy, activation of the DNA-damage response/DNA-dependent protein kinase pathway increased TFRC and ACSL4 while reducing GPX4 and SLC7A11, resulting in malondialdehyde and 4-hydroxynonenal accumulation and cardiac microvascular injury [132]. MAP4K4-mediated S-nitrosylation of Drp1 further suppressed GPX4 and aggravated cardiac microvascular endothelial ferroptosis [342].

Iron–lipid balance also influences pancreatic and metabolic immune compartments. Human islets exposed to ferroptosis-inducing agents showed impaired viability and insulin-secretory function [343]. Islet-resident macrophages use an SLC7A11-dependent antioxidant programme to preserve β-cell physiology [344], whereas CXCL16-dependent scavenging of oxidised lipids by islet macrophages promotes pathogenic CD8-positive T-cell differentiation in autoimmune diabetes [345]. Deletion of H-ferritin in macrophages alleviated high-fat-diet-induced obesity and insulin resistance [346]. These pancreatic and systemic metabolic studies provide a broader inflammatory context but do not constitute direct evidence of vascular injury.

Nicorandil reduced cardiac microvascular ferroptosis in experimental diabetic cardiomyopathy through mitochondria-associated AMP-activated protein kinase (AMPK)–Parkin–ACSL4 signalling [347]. This finding supports the biological relevance of ferroptotic injury in diabetic microvasculature but does not establish systemic iron chelation as a treatment for diabetic angiopathy. The general execution of ferroptosis is described in Section 3.2.

#### 4.7.2. Cu: Endothelial Redox Imbalance and Locally Controlled Vascular Repair

In hyperglycaemic cerebral endothelial cells, Cu/Zn-superoxide dismutase expression and activity were altered together with reduced endothelial nitric oxide synthase and increased endothelin-1 [348]. These changes demonstrate disruption of the Cu/Zn-superoxide-dismutase-associated redox system under hyperglycaemic conditions. They do not directly demonstrate tissue copper accumulation or show that copper supplementation restores endothelial function. Accordingly, total serum or tissue copper measurements should not be interpreted as direct measures of the chemically reactive labile or exchangeable copper pool responsible for redox toxicity.

Copper-containing hydrogels, metal–organic frameworks, and nanozymes have shown antibacterial, reactive-oxygen-species-modulating, and pro-angiogenic effects in experimental diabetic wound models [199,349,350,351,352]. Reported responses include increased hypoxia-inducible factor-1α, VEGF, and endothelial nitric oxide synthase expression, enhanced endothelial migration, and promotion of reparative macrophage phenotypes [199,349]. These systems use controlled local delivery and should not be interpreted as evidence that systemic copper exposure is beneficial in diabetic angiopathy. Their material design and therapeutic applications are discussed in Section 5.3.

## 5. Therapeutic Strategies Based on Modulating Metal Metabolism and Antioxidant Defence

Therapeutic responses to metal-directed interventions vary with cell type, disease phase, exposure route, and the metal pool being targeted. For example, haem oxygenase-1 exerts different effects in VSMCs and macrophages during abdominal aortic aneurysm development; acute zinc accumulation aggravates neuronal injury, whereas zinc administration during the recovery phase can support post-ischaemic repair; and both chronic copper deficiency and acute mitochondrial copper accumulation impair cardiac function through distinct mechanisms [259,262,283,297,332,333,334,335]. These findings argue against indiscriminate chelation or supplementation and favour interventions matched to a defined pathological context.

### 5.1. Clinical Evidence and Limitations of Metal Chelation

Deferoxamine (DFO) chelates labile iron and can reduce iron-dependent oxidative injury [353]. DFO also stabilises hypoxia-inducible factor-1α and increases VEGF and stromal cell-derived factor-1α signalling. Hydrogels, microneedles, scaffolds, and nanoparticles have therefore been developed to prolong local DFO exposure and support angiogenesis in wound- and bone-repair models [354,355,356,357,358,359,360]. However, these studies predominantly assess preclinical tissue-repair or surrogate angiogenic endpoints and do not establish improvement in major cardiovascular or neurological outcomes.

Clinical translation in ICH has been less conclusive. In the phase 2 trial of DFO in patients with ICH, DFO initiated within 24 h after ICH was acceptably safe but did not improve the prespecified 90-day functional outcome. A modified Rankin Scale score of 0–2 was observed in 34% of DFO-treated participants and 33% of placebo-treated participants, and the prespecified analysis indicated futility for proceeding to a phase 3 trial on the assumption of a clinically substantial 90-day benefit [361]. These findings do not support routine DFO treatment after ICH and indicate that administration within 24 h did not, by itself, identify an effective clinical therapeutic window. Future trials will need to determine whether treatment response depends on patient selection, haematoma-associated iron burden, and evidence of target engagement.

Broader cardiovascular evidence for chelation is also inconsistent. The original Trial to Assess Chelation Therapy reported an 18% relative reduction in its composite cardiovascular endpoint after edetate disodium-based infusions in patients with previous myocardial infarction [362]. However, the Trial to Assess Chelation Therapy 2 (TACT2) did not replicate this benefit in patients with diabetes and previous myocardial infarction. Although edetate disodium-based chelation reduced median blood lead concentrations by 61%, the primary cardiovascular endpoint was similar between the chelation and placebo groups (adjusted hazard ratio, 0.93; 95% confidence interval, 0.76–1.16) [363]. Thus, effective reduction of a circulating metal biomarker does not necessarily translate into fewer cardiovascular events.

Tetrathiomolybdate (TM) reduces copper availability and also exhibits redox-active and hydrogen-sulphide-releasing properties [364]. Its biological effects are potentially context dependent because suppression of copper-dependent angiogenesis may be advantageous in pathological proliferation but undesirable during ischaemic tissue repair. The evidence reviewed here is derived mainly from Wilson disease, copper toxicity, and preclinical inflammatory or reperfusion models; cardiovascular outcome benefits have not been established.

### 5.2. Trace-Element Supplementation: Evidence, Route, and Risk of Overcorrection

Although oxidative stress is central to cardiovascular and metabolic disease, conventional antioxidant supplementation has frequently failed to improve cardiovascular outcomes [365,366]. ROS also participate in adaptive signalling, and non-targeted antioxidant treatment may blunt physiological responses or fail to reach the subcellular sites at which pathological oxidants are generated [365,367,368]. The effects of vitamin E likewise vary by disease context and may involve mechanisms beyond direct radical scavenging [366,369]. These limitations support targeting defined sources of redox dysregulation rather than indiscriminate systemic scavenging.

Iron supplementation illustrates the importance of formulation and biological availability. In the IRONOUT-HF randomised trial, high-dose oral iron polysaccharide minimally increased iron stores and did not improve peak oxygen uptake, six-minute walk distance, natriuretic peptide concentrations, or health status in patients with iron-deficient heart failure with reduced ejection fraction [370]. Participants with higher baseline hepcidin concentrations showed a weaker biochemical response to oral iron, supporting restricted intestinal absorption and iron mobilisation as contributors to the neutral result. The neutral result was specific to oral iron and should not be generalised to intravenous formulations. In AFFIRM-AHF, intravenous ferric carboxymaltose initiated after clinical stabilisation from an acute heart-failure episode reduced subsequent heart-failure hospitalisations but had no apparent effect on cardiovascular mortality [371]. These contrasting results indicate that the therapeutic window for iron repletion depends not only on the presence of iron deficiency but also on formulation, absorption, and the clinical phase at which treatment is initiated.

Zinc supplementation also requires distinction between local delivery and systemic exposure. Zinc released from experimental implants enhanced endothelial viability, neurovascularisation, reparative macrophage polarisation, and osseointegration [372]. By contrast, observational data in diabetes associated serum zinc and the copper-to-zinc ratio with different macrovascular and microvascular complications in sex-dependent patterns [373]. Local biomaterial effects and circulating observational associations do not define a universally beneficial serum zinc concentration or establish that unselected systemic zinc supplementation prevents vascular disease. Clinical studies should therefore distinguish confirmed deficiency from altered distribution and account for sex and metabolic context.

### 5.3. Nanomedicine and Targeted Delivery Systems

Addressing the complex pathophysiology of oxidative stress and metal ion homeostasis imbalance in cardiovascular and cerebrovascular diseases, nanomedicine offers a precise dual-action strategy. Through the design of intelligent nanocarriers, it not only enables targeted drug delivery to plaques or ischaemic sites but also synergistically combines metal chelators with antioxidants to achieve enhanced therapeutic effects.

In the treatment of ischaemia-reperfusion injury (IRI), constructing multi-level nanoplatforms that combine ion regulation with ROS scavenging has emerged as a cutting-edge approach. For instance, the bionic nanomedicine strontium-substituted hexacyanoferrate (SrHCF), a Prussian blue analogue, has been engineered for treating cerebral I/R injury. This carrier exhibits multiple synergistic mechanisms: first, its Fe-CN-Sr coordination network efficiently traps excess endogenous ferrous ions (Fe^2+^), inhibiting the Fenton reaction and triggering lattice restructuring to generate Prussian blue (PB) with enhanced antioxidant activity; second, it exhibits SOD-like and CAT-like activities that scavenge ROS. Finally, the concurrent release of Sr^2+^ effectively antagonises Ca^2+^ overload. This integrated strategy of ‘ferrous chelation-antioxidation-calcium antagonism’ significantly suppresses neuronal ferroptosis and inflammatory storms [374].

For the microenvironments of myocardial infarction (MI) and stroke, nanocarriers have been endowed with more sophisticated enzyme-mimetic and gas delivery capabilities. Prussian blue nanoenzymes (PBNz) have been extensively employed for scavenging ROS in ischaemic myocardium and inducing macrophage polarisation towards the anti-inflammatory M2 phenotype, owing to their exceptional multienzyme-mimetic activity (SOD/CAT/peroxidase) [375]. To address the challenge of NO therapy readily generating toxic peroxynitrite (ONOO^−^) in oxidative environments, researchers developed Prussian blue nanolattices embedded with biliverdin and NO donors. Leveraging the ROS-scavenging properties of PB and biliverdin, this approach ensures NO exerts its pro-angiogenic effects without ONOO^−^ generation [376]. Furthermore, addressing the BBB impediment, bionic nanoscale systems mimicking neutrophil membranes or cerium oxide nanoparticles modified with Angiopep-2 effectively traverse the BBB to target ischaemic brain tissue. These approaches salvage damaged neurons by scavenging ROS and replenishing energy metabolism substrates such as ATP/NADPH [377,378].

In the treatment of atherosclerosis and vascular calcification, smart nanomedicines stabilise plaques by regulating lipid metabolism and metal deposition. A multifunctional nanomedicine co-delivers Zn^2+^, epigallocatechin gallate (EGCG), and CpG oligonucleotides via a metal-organic framework shell. Specifically, Zn^2+^ promotes intracellular lipid degradation, EGCG acts as an antioxidant and facilitates cholesterol efflux, while CpG enhances macrophage phagocytosis. This tripartite synergy achieves an anti-atherosclerotic cascade effect: ‘phagocytosis-lipid degradation-cholesterol efflux’ [379]. For vascular calcification, poly(succinimidyl)imide nanoparticles (PSI NPs) were developed as a prodrug carrier for polyaspartic acid (a potent calcium chelator), simultaneously encapsulating the hydrophobic antioxidant curcumin. Under physiological conditions, this system slowly decomposes to release calcium chelators that inhibit calcification. Concurrently released curcumin effectively reduces macrophage ROS levels and suppresses osteogenic differentiation in VSMCs, achieving synergistic anti-calcification and antioxidant therapy [380]. Furthermore, black phosphorus nanosheets (BPNSs) and cyclodextrin-based broad-spectrum ROS-scavenging nanoparticles (TPCD NPs) have demonstrated significant potential for plaque stabilisation through ROS clearance and delivery of anti-inflammatory mediators such as Resolvin D1 [381,382].

Despite their mechanistic appeal, these nanoplatforms remain preclinical. Direct comparison across studies is limited by differences in disease models, dosing metrics, treatment timing, and predominantly short-term endpoints. Moreover, multifunctional particles frequently combine metal chelation, reactive-oxygen-species scavenging, ion release, enzyme-mimetic activity, and delivery of additional bioactive compounds, making it difficult to identify which component accounts for the observed benefit. Clinical translation will require standardised assessment of biodistribution, metal-release kinetics, long-term safety, and efficacy against appropriate standard-of-care comparators.

### 5.4. Clinically Available Metal-Related Biomarkers and Their Current Utility

Iron-related measurements are the most established metal biomarkers in cardiovascular practice. Serum ferritin estimates iron stores, whereas transferrin saturation (TSAT) reflects circulating iron availability. In heart failure, current clinical criteria define iron deficiency as ferritin <100 μg/L or ferritin of 100–299 μg/L with TSAT <20%, and the two measurements are therefore interpreted together [383]. Ferritin is also an acute-phase reactant and may increase during inflammation, liver injury, or acute illness, reducing the reliability of an isolated value. Cardiovascular magnetic resonance T2* provides a clinically available tissue-specific assessment of myocardial iron in iron-overload disorders [384]. However, neither circulating iron indices nor myocardial T2* directly measures the labile intracellular iron pool or ferroptotic activity.

Clinically available copper-related tests include serum or plasma ceruloplasmin, total serum copper, and 24-h urinary copper excretion. These measurements are used principally in the diagnosis and longitudinal assessment of systemic copper disorders, especially Wilson disease, and should be interpreted in combination rather than individually. The updated clinical practice guidelines on Wilson disease also incorporate relative exchangeable copper, although access and assay standardisation remain more limited [385]. In cardiovascular and cerebrovascular disorders, serum copper and ceruloplasmin may provide associative or prognostic information, but disease-specific thresholds for routine risk stratification or treatment selection have not been established.

Plasma or serum zinc is the most accessible laboratory measure of zinc status, but its interpretation is sensitive to fasting status, time of sampling, inflammation, infection, albumin concentration, and recent dietary intake [386,387]. A low circulating zinc concentration may therefore reflect nutritional deficiency, acute redistribution, or an inflammatory response. Measurements such as the serum copper-to-zinc ratio have shown observational associations with vascular complications, but a universally accepted cardiovascular or cerebrovascular decision threshold is not currently available [373]. Thus, zinc-related measurements are most informative when interpreted with the clinical, nutritional, and inflammatory context rather than as isolated indicators of intracellular zinc availability.

### 5.5. Genetic Variability in Metal Homeostasis and Treatment Response

Genetic variation can modify baseline metal status and individual susceptibility, but genotype does not invariably predict clinical disease. The *HFE* C282Y variant illustrates this incomplete penetrance: in a large prospective cohort, iron-overload-related disease developed in a substantial proportion of male C282Y homozygotes but was uncommon in female homozygotes, indicating that sex and other modifiers influence the phenotypic consequences of the same iron-loading genotype [388]. Variation in the hepcidin regulator *TMPRSS6* may also affect treatment exposure. In a stable-isotope study, women carrying the rs855791 TT genotype had lower fractional absorption of orally administered iron than CC carriers [389]. These findings suggest that inherited regulation of the hepcidin–ferroportin axis can contribute to interindividual variation in iron status and oral supplementation response. However, routine *TMPRSS6* genotyping has not been validated for selecting oral versus intravenous iron treatment in cardiovascular disease.

Genetic effects are also evident in copper and zinc metabolism. Biallelic pathogenic *copper-transporting P-type ATPase 7B (ATP7B)* variants cause defective biliary copper excretion, and a retrospective Wilson disease cohort associated loss-of-function variants with poorer long-term outcomes among patients with chronic liver disease receiving chelation [390]. This finding supports genotype-informed risk assessment, although it does not establish that *ATP7B* genotype alone should determine the choice between chelation and zinc therapy. *ATP7A*-related Menkes disease and occipital horn syndrome provide a complementary human context: pathogenic *ATP7A* variants impair copper delivery to secretory cuproenzymes, including lysyl oxidase, and are associated with connective-tissue abnormalities [391]. These rare inherited disorders support the biological importance of ATP7A-dependent copper delivery but do not establish the same mechanism in common cardiovascular disease. For zinc, rare protein-truncating variants in *SLC30A8*, which encodes the islet zinc transporter ZnT8, were associated with a 65% lower risk of type 2 diabetes [392]. This human genetic evidence identifies zinc transport as a determinant of metabolic susceptibility and a potential therapeutic target, but it should not be interpreted as evidence that systemic zinc restriction or supplementation will reproduce the genetic effect. At present, genetic testing is most informative for diagnosing or stratifying inherited metal disorders; its value for guiding metal-directed treatment in common cardiovascular and cerebrovascular diseases remains to be established.

### 5.6. Therapeutic Windows for Chelation and Supplementation

The therapeutic window for a metal-directed intervention should be defined by the direction of metal dysregulation, the disease phase, and evidence that the targeted metal pool is biologically relevant. In acute haemorrhagic or reperfusion injury, chelation is mechanistically most plausible during the interval in which haemolysis, cellular damage, or impaired export increases a labile metal pool before irreversible tissue injury is established. However, the phase 2 DFO trial demonstrates that treatment based on clock time alone is insufficient: DFO initiated within 24 h after ICH did not improve the prespecified 90-day functional outcome [361]. Acute supplementation also requires caution because a reduced circulating concentration may reflect redistribution rather than true deficiency, and increasing metal availability during an oxidative burst may aggravate redox injury.

The distinction between injury and recovery phases is illustrated by experimental cerebral ischaemia. Zinc chelation during acute injury reduced zinc-associated inflammatory and endoplasmic-reticulum stress, whereas zinc administration during the recovery phase promoted astrocyte-dependent angiogenesis and neurological recovery [295,296,297]. These preclinical findings support phase-specific intervention but do not define a human dose or treatment window. In chronic disease, treatment should instead be based on persistent, clinically validated evidence of overload or deficiency and accompanied by repeated monitoring. Prolonged non-selective chelation may deplete metals required for mitochondrial, antioxidant, or repair enzymes, whereas supplementation without confirmed deficiency risks overcorrection. The contrasting results of IRONOUT-HF and AFFIRM-AHF further show that oral absorption, intravenous delivery, clinical stability, and the selected endpoint can determine whether iron repletion produces measurable benefit [370,371]. Thus, chelation and supplementation should not be regarded as universally opposing strategies; where validated biomarkers are available, treatment eligibility should be guided by ferritin and transferrin saturation (TSAT) for iron deficiency or myocardial T2* for cardiac iron overload, together with disease phase and predefined reassessment criteria. The translational status of representative metal-directed therapeutic strategies is summarized in Table 3.

## 6. Conclusions and Testable Perspectives

The onset and progression of cardiovascular and cerebrovascular diseases are not attributable to a single factor, but rather represent a complex pathological process involving the intertwined and mutually reinforcing effects of metal ion metabolic imbalance and oxidative stress. This review summarizes the physiological and pathological roles of iron, copper, and zinc and examines mechanisms through which their dyshomeostasis may contribute to cardiovascular and cerebrovascular injury.

In selected pathological settings, expansion of labile Fe or Cu pools can promote Fenton-type redox chemistry and contribute to oxidative modification of DNA and proteins, including 8-OHdG formation and CaMKII oxidation [108,125].

Regulated cell-death pathways are among the downstream consequences of metal dyshomeostasis. Ferroptosis, driven by iron-dependent lipid peroxidation, has been implicated in I/R injury, myocardial infarction, and atherosclerotic plaque rupture. Its occurrence involves the collapse of the GPX4-GSH axis, NCOA4-mediated ferritinophagy, and ACSL4-driven lipid remodelling [12,18]. Conversely, experimental studies indicate that cuproptosis can disrupt mitochondrial TCA-cycle function through FDX1-associated lipoylated-protein aggregation and may contribute to cardiac injury; its importance in human cardiovascular and cerebrovascular diseases remains to be established [187].

Zinc dyshomeostasis also has context-dependent pathological effects. Within vascular endothelium, zinc deficiency can promote eNOS decoupling and impair Nrf2-associated antioxidant defence [254]; in ischaemic stroke, acute zinc redistribution can promote mitochondrial dysfunction and ROS amplification, contributing to delayed neuronal injury [125].

Despite significant advances in our understanding of the relationship between metal ions and cardiovascular disease, several key challenges remain to be overcome before achieving clinical translation:

Currently available blood tests provide clinically useful information about systemic iron, copper, and zinc status but do not directly quantify the labile or subcellular metal pools that mediate oxidative injury. A major translational priority is therefore to validate tissue- and compartment-specific biomarkers that complement established clinical measurements.

A first testable hypothesis is that local iron burden, rather than time from symptom onset alone, identifies patients with ICH who are most likely to benefit from iron chelation. This hypothesis could be examined prospectively by combining serial iron-sensitive brain imaging with perihaematomal oedema, circulating iron indices, and functional outcomes in a biomarker-stratified chelation trial [307,361]. It would be supported if baseline or evolving local iron burden improved outcome prediction and showed a treatment-by-biomarker interaction beyond that provided by treatment timing alone.

A second hypothesis is that zinc-directed therapy after cerebral ischaemia has a phase-dependent directional switch: reducing labile Zn^2+^ during acute injury will limit neuronal and neurovascular damage, whereas restoring controlled zinc availability during recovery will enhance angiogenesis and functional repair [295,296,297]. This hypothesis should be tested within the same experimental system by varying intervention timing while measuring labile zinc, acute infarct and blood–brain barrier injury, and later angiogenic and neurological outcomes. It would be supported if chelation and supplementation produced benefit in distinct, non-overlapping phases rather than exerting the same effect irrespective of timing.

A third hypothesis is that mitochondrial copper accumulation, rather than total myocardial copper content, is the more informative determinant of cuproptosis-associated myocardial ischaemia–reperfusion injury. This could be tested by comparing total and mitochondrial copper measurements with ATP7A activity, aggregation of lipoylated mitochondrial proteins, loss of iron–sulphur cluster proteins, and myocardial injury after compartment-specific or non-selective copper modulation [259,260]. The hypothesis would be supported if mitochondrial copper measurements predicted injury and treatment response more accurately than total copper and if mitochondria-directed intervention provided benefit at an exposure that avoided systemic copper depletion. Targeted nanocarriers may provide one experimental means of testing this compartment-specific intervention principle, but their effects should be compared with composition-matched non-targeted controls.

Collectively, these hypotheses shift future research from broadly modifying systemic metal concentrations towards identifying the relevant metal pool, disease phase, and responsive patient subgroup. Their evaluation will require prospective biomarker-defined studies, direct measurement of target engagement, and comparisons with appropriate non-targeted or standard-of-care controls. Such an approach will determine whether metal-directed interventions can progress from mechanistic plausibility to reproducible clinical benefit.

## Figures and Tables

**Table 1:** Comparative physiological functions and pathological characteristics of iron, copper and zinc in the cardiovascular and cerebrovascular systems.

Metal	Carriers/Regulators	Physiological Function	Pathological Mechanism	Consequences	References
Fe	Carrier: Transferrin, Ferritin. Regulation: IRPs/IRE, Hepcidin.	Mitochondrial respiration: Fe–S proteins in respiratory-chain complexes I–III and haem iron in mitochondrial cytochromes. Synthesis: Haem and Fe–S clusters synthesis.	Fenton reaction: Hydroxyl radical burst, DNA 8-OHdG modification. Ferroptosis: Membrane lipid peroxidation, GPX4 depletion.	Myocardial I/R injury. Atherosclerotic plaque haemorrhage. Neuronal death following cerebral infarction	[12,24,32]
Cu	Carrier: Ceruloplasmin, Albumin. Regulation: ATP7A and ATP7B, CTR1.	Mitochondrial respiration: Copper-containing catalytic centres in cytochrome c oxidase (Complex IV). Antioxidant: Cu/Zn-SOD active centre. Matrix cross-linking: LOX-mediated elastin/collagen cross-linking. Angiogenesis: Promoting VEGF expression.	Cuproptosis: Targeting Mitochondrial TCA Cycle (DLAT Oligomerisation). Oxidative catalysis: Catalysing LDL oxidation (ox-LDL).	AAA. Hypertrophic cardiomyopathy/Heart failure. Diabetic vascular complications.	[13,65,66]
Zn	Carrier: Albumin, Macroglobulin. Regulation: ZnT, ZIP, MT (metallothionein).	Anti-inflammatory: Activation of A20 inhibits NF-κB. Antioxidant: Inhibition of Keap1–Cul3-mediated Nrf2 ubiquitination. Enzyme stability: maintaining the dimeric conformation of eNOS.	eNOS decoupling: conversion into ROS generators, nitrosative stress. Excitotoxicity: Mitochondrial zinc overload induces ROS cycling.	Hypertension/Endothelial Dysfunction. Ischemic stroke (delayed death). vascular calcification.	[9,82,102]

Abbreviations: 8-OHdG, 8-hydroxy-2′-deoxyguanosine; A20, tumour necrosis factor alpha-induced protein 3; AAA, abdominal aortic aneurysm; ATP7A/ATP7B, copper-transporting P-type ATPases 7A and 7B; CTR1, copper transporter 1; Cul3, cullin 3; Cu/Zn-SOD, copper/zinc superoxide dismutase; DLAT, dihydrolipoamide S-acetyltransferase; DNA, deoxyribonucleic acid; eNOS, endothelial nitric oxide synthase; Fe–S, iron–sulphur; GPX4, glutathione peroxidase 4; I/R, ischaemia–reperfusion; IRE, iron-responsive element; IRP(s), iron regulatory protein(s); Keap1, Kelch-like ECH-associated protein 1; LDL/ox-LDL, low-density lipoprotein/oxidised low-density lipoprotein; LOX, lysyl oxidase; MT, metallothionein; NF-κB, nuclear factor kappa B; Nrf2, nuclear factor erythroid 2-related factor 2; ROS, reactive oxygen species; TCA, tricarboxylic acid; VEGF, vascular endothelial growth factor; ZIP, Zrt- and Irt-like protein family; ZnT, zinc transporter family.

**Table 2:** Comparison of ferroptosis and cuproptosis as metal-associated regulated cell-death mechanisms.

Feature	Ferroptosis	Cuproptosis	References
Core mechanism	Iron dependence and phospholipid peroxidation	Copper overload in cells dependent on mitochondrial respiration.	[115]
Cellular context	Phospholipid membrane	Mitochondrial TCA-cycle machinery.	[116]
Key molecular components	GPX4-GSH axis, ACSL4, NCOA4	FDX1, DLAT/DLST, and LIAS-dependent protein lipoylation.	[12]
Biochemical/Morphological Characteristics	Accumulation of membrane lipid peroxides; mitochondrial shrinkage and increased membrane density.	Aggregation of lipoylated proteins, loss of Fe-S cluster proteins, and proteotoxic stress.	[117]

Abbreviations: GSH, glutathione; GPX4, glutathione peroxidase 4; ACSL4, acyl-CoA synthetase long-chain family member 4; NCOA4, nuclear receptor coactivator 4; FDX1, ferredoxin 1; DLAT, dihydrolipoamide S-acetyltransferase; DLST, dihydrolipoamide S-succinyltransferase; Fe–S, iron–sulphur; TCA, tricarboxylic acid; LIAS, lipoyl synthase.

**Table 3:** Evidence-stratified summary of metal-directed therapeutic strategies and their translational status.

Intervention	Disease/Model	Evidence	Main Outcome	Limitations/ Status	References
Copper-directed therapy	Wilson disease	Clinical practice/guideline	Controls pathological systemic copper accumulation	Disease-specific; cannot be directly extrapolated to CCVDs	[385]
Deferoxamine (iron chelation)	Intracerebral haemorrhage	Phase 2 RCT	Acceptably safe, but did not improve the prespecified 90-day functional outcome	Clinical benefit not established; optimal patient selection remains uncertain	[361]
Edetate disodium-based chelation	Previous myocardial infarction	RCTs	TACT suggested benefit, but TACT2 did not replicate it	Cardiovascular benefit remains uncertain despite reduction of blood lead	[362,363]
Iron repletion	Iron-deficient heart failure	RCTs	Oral iron was neutral; intravenous ferric carboxymaltose reduced heart-failure hospitalisations without clear mortality benefit	Effects depend on formulation, absorption, and clinical context	[370,371]
Zinc status/supplementation	Diabetes-related vascular complications	Human observational	Circulating zinc and Cu/Zn ratio showed context-dependent associations with vascular complications	Does not establish benefit of supplementation or a therapeutic zinc threshold	[373]

Abbreviations: CCVDs, cardiovascular and cerebrovascular diseases; Cu/Zn, copper-to-zinc ratio; RCT(s), randomised controlled trial(s); TACT, Trial to Assess Chelation Therapy; TACT2, Trial to Assess Chelation Therapy 2.

## Data Availability

No new data were created or analysed in this study. Data sharing is not applicable to this article.

## Abbreviations

Abbreviation	Definition	Abbreviation	Definition
8-OHdG	8-hydroxy-2′-deoxyguanosine	A20	tumour necrosis factor alpha-induced protein 3 (TNFAIP3)
AAA	abdominal aortic aneurysm	ACSL4	acyl-CoA synthetase long-chain family member 4
AMPK	AMP-activated protein kinase	Ang II	angiotensin II
APJ	apelin receptor	ARE	antioxidant response element
ATP7A/ATP7B	copper-transporting P-type ATPases 7A and 7B	BBB	blood–brain barrier
CaMKII	calcium/calmodulin-dependent protein kinase II	CAT	catalase
CCVDs	cardiovascular and cerebrovascular diseases	cGAS–STING	cyclic GMP–AMP synthase–stimulator of interferon genes
cGMP	cyclic guanosine monophosphate	CTR1	copper transporter 1
Cu-MOF	copper-based metal–organic framework	Cul3	cullin 3
CVD	cardiovascular disease	DFO	deferoxamine
DLAT	dihydrolipoamide S-acetyltransferase	Drp1	dynamin-related protein 1
ECM	extracellular matrix	eNOS	endothelial nitric oxide synthase
FDX1	ferredoxin 1	Fe–S	iron–sulphur
FENDRR	FOXF1 adjacent non-coding developmental regulatory RNA	FTH1	ferritin heavy chain 1
GCLC	glutamate-cysteine ligase catalytic subunit	GPX4	glutathione peroxidase 4
GSH	glutathione	GUCY1A1	guanylate cyclase 1 soluble subunit alpha 1
HIF-1α	hypoxia-inducible factor-1α	HIF-2α	hypoxia-inducible factor-2α
HO-1	haem oxygenase 1	I/R	ischaemia–reperfusion
ICH	intracerebral haemorrhage	IRE	iron-responsive element
IRP	iron regulatory protein	IRI	ischaemia–reperfusion injury
Keap1	Kelch-like ECH-associated protein 1	LDL	low-density lipoprotein
LIAS	lipoyl synthase	LIP	labile iron pool
LOX	lysyl oxidase	LPC	lysophosphatidylcholine
Lp-PLA2	lipoprotein-associated phospholipase A2	MAPK	mitogen-activated protein kinase
MMP	matrix metalloproteinase	MOF	metal–organic framework
MT	metallothionein	NADPH	reduced nicotinamide adenine dinucleotide phosphate
NCOA4	nuclear receptor coactivator 4	NF-κB	nuclear factor kappa B
NLRP3	NLR family pyrin domain-containing 3	NO	nitric oxide
NQO1	NAD(P)H quinone dehydrogenase 1	Nrf2	nuclear factor erythroid 2-related factor 2
OXPHOS	oxidative phosphorylation	ox-LDL	oxidised low-density lipoprotein
PB	Prussian blue	PKG	cGMP-dependent protein kinase
PKM2	pyruvate kinase M2	PUFA	polyunsaturated fatty acid
ROS	reactive oxygen species	SENP2	small ubiquitin-like modifier-specific protease 2
SLC7A11	solute carrier family 7 member 11	SMLC	S-methyl-L-cysteine
SOD	superoxide dismutase	SrHCF	strontium-substituted hexacyanoferrate
SSP	serine synthesis pathway	SUMO	small ubiquitin-like modifier
TCA	tricarboxylic acid	TfR1	transferrin receptor 1
TM	tetrathiomolybdate	TNF-α	tumour necrosis factor-α
TNFAIP3	tumour necrosis factor alpha-induced protein 3	UTR	untranslated region
VDAC1	voltage-dependent anion channel 1	VEGF	vascular endothelial growth factor
VEGFR2	vascular endothelial growth factor receptor 2	VSMC	vascular smooth muscle cell
YTHDF2	YTH N6-methyladenosine RNA-binding protein 2	ZIP	Zrt- and Irt-like protein family
ZnT	zinc transporter family		
